# Mucin acts as a nutrient source and a signal for the differential expression of genes coding for cellular processes and virulence factors in *Acinetobacter baumannii*

**DOI:** 10.1371/journal.pone.0190599

**Published:** 2018-01-08

**Authors:** Emily J. Ohneck, Brock A. Arivett, Steven E. Fiester, Cecily R. Wood, Maeva L. Metz, Gabriella M. Simeone, Luis A. Actis

**Affiliations:** Department of Microbiology, Miami University, Oxford, OH, United States of America; University of Hyderabad, INDIA

## Abstract

The capacity of *Acinetobacter baumannii* to persist and cause infections depends on its interaction with abiotic and biotic surfaces, including those found on medical devices and host mucosal surfaces. However, the extracellular stimuli affecting these interactions are poorly understood. Based on our previous observations, we hypothesized that mucin, a glycoprotein secreted by lung epithelial cells, particularly during respiratory infections, significantly alters *A*. *baumannii*’s physiology and its interaction with the surrounding environment. Biofilm, virulence and growth assays showed that mucin enhances the interaction of *A*. *baumannii* ATCC 19606^T^ with abiotic and biotic surfaces and its cytolytic activity against epithelial cells while serving as a nutrient source. The global effect of mucin on the physiology and virulence of this pathogen is supported by RNA-Seq data showing that its presence in a low nutrient medium results in the differential transcription of 427 predicted protein-coding genes. The reduced expression of ion acquisition genes and the increased transcription of genes coding for energy production together with the detection of mucin degradation indicate that this host glycoprotein is a nutrient source. The increased expression of genes coding for adherence and biofilm biogenesis on abiotic and biotic surfaces, the degradation of phenylacetic acid and the production of an active type VI secretion system further supports the role mucin plays in virulence. Taken together, our observations indicate that *A*. *baumannii* recognizes mucin as an environmental signal, which triggers a response cascade that allows this pathogen to acquire critical nutrients and promotes host-pathogen interactions that play a role in the pathogenesis of bacterial infections.

## Introduction

*Acinetobacter baumannii* causes several types of severe infections in compromised individuals [1, 2]. This opportunistic pathogen is responsible for over 10% of infections that occur in intensive care units and is capable of causing respiratory tract infections, necrotizing fasciitis, and infections associated with intravascular devices, ulcers, surgical sites and severe wounds [3–10]. Patients infected with *A*. *baumannii* have a mortality rate ranging from 20% to 50%, which may be attributed to cellular functions such as capsule production, biofilm formation, the expression of a variety of nutrient and iron acquisition pathways, the ability to cope with environmental stress, and the acquisition and expression of multidrug resistance (MDR) genes [11]. This problem is further compounded by our limited understanding of the mechanisms by which *A*. *baumannii* interacts with the human host. Due to the increasing number of MDR *A*. *baumannii* nosocomial isolates and the wide range of cellular mechanisms contributing to *A*. *baumannii* virulence, different therapeutic approaches are required to control the severe infections this pathogen causes in humans [12].

Ventilator-associated pneumonia (VAP) caused by *A*. *baumannii* is increasing in prevalence, particularly in patients staying in intensive care units for extended periods of time [13, 14]. Patients with VAP commonly experience hyper-secretion of mucus in the respiratory tract, which normally acts as a barrier against pathogens; however, overproduction of mucus in the respiratory tract can cause patients to become susceptible to infection with opportunistic pathogens [14, 15]. A large component of mucus is mucin, a heavily glycosylated protein produced by epithelial cells that provides protection from the external environment, protects against activation of the inflammatory response at the epithelial barrier, aids in tissue repair, and participates in both lubrication and cell-to-cell signaling [16]. However, increased amounts of mucin, such as those commonly associated with VAP, decrease host resistance to infection by inhibition of the host complement pathway through the inhibition of properdin, also known as Factor P [17, 18]. The heavy glycosylation of mucins combined with their ability to suppress the host inflammatory response provide a protected, nutrient-rich environment, which facilitates colonization by opportunistic pathogens as previously described for *Pseudomonas aeruginosa* [15, 19–21]. *A*. *baumannii* is often associated with nosocomial respiratory infections in immunocompromised individuals, but it remains unknown how this pathogen interacts with mucin in the host environment. Therefore, *A*. *baumannii*’s interaction with mucin merits further study.

Previous studies have used A549 human alveolar epithelial cells as a model to examine host-pathogen interactions because they closely represent the host environment [22, 23]. Interestingly, A549 cells secrete a surfactant that mimics the surfactant produced by lung epithelial cells, of which a major component is mucin [24–27]. In addition, previous studies have demonstrated the ability of *A*. *baumannii* to attach to host cells and have suggested that *A*. *baumannii* expresses multiple mechanisms of adherence to both biotic and abiotic surfaces [22, 23]. However, the nature of the signal or signals to which *A*. *baumannii* responds in order to regulate the expression of attachment mechanisms or other virulence factors in response to abiotic or biotic surfaces remains largely unknown. Due to its role in regulation of the inflammatory response, mucin has been used to decrease host resistance and therefore increase the apparent virulence of pathogens in several studies [17, 28, 29]. This effect has also been demonstrated with *A*. *baumannii* infections in mice [30]. Although mucin is known to aid host innate immunity, pathogens also possess several mechanisms to evade the protective properties of mucin [18].

Since the role of mucin in *A*. *baumannii* pathogenicity has not been described, this study aims to determine how *A*. *baumannii* ATCC 19606^T^ responds to mucin and whether this host product is recognized as a signal for increased virulence. In this study we observed that the presence of porcine gastric mucin (PGM), at a biologically relevant concentration in a nutrient limited medium, elicits a global cellular response that is consistent with the ability of *A*. *baumannii* to use this glycoprotein as a nutrient source while promoting the differential expression of virulence associated genes. Taken together, these observations indicate that mucin plays a critical role in host responses to bacterial infections and affects bacterial responses that play a critical role in the pathogenesis of infections.

## Materials and methods

### Bacterial strains, media, and culture conditions

*A*. *baumannii* ATCC 19606^T^ was purchased from the American Type Culture Collection (ATCC) and porcine gastric mucin type III was obtained from Sigma-Aldrich. *A*. *baumannii* ATCC 19606^T^ was stored as Luria-Bertani (LB) broth/glycerol stocks at -80°C [31]. *Escherichia coli* MG1655 was obtained from F. Blattner, University of Wisconsin, USA. Cultures on LB agar were used to inoculate either LB broth [31] or swimming broth (SB, 1% tryptone, 0.5% NaCl), which were incubated overnight (12 to 14 h) at 37°C with shaking at 200 rpm. Swimming agar (SA) plates were made by adding 0.3% agarose to SB. Solutions containing mucin were sterilized by autoclaving as described previously [15, 19–21]. A549 human alveolar epithelial cells, which were obtained from Dr. E. Lafontaine, College of Veterinary Medicine, University of Georgia, USA, were cultured in Dulbecco’s modified Eagle’s medium (DMEM) supplemented with 10% heat-inactivated fetal bovine serum, ampicillin and streptomycin at 37°C in the presence of 5% CO_2_, as previously described [32].

### Bacterial growth analysis

Bacterial growth curves were performed by inoculating SB, SB supplemented with 0.5% mucin (SB+M) or mucin broth (MB, 0.5% mucin in dH_2_O) with a 1:100 dilution of an overnight culture grown in SB with an OD_600_ ranging between 1.40 and 1.50. Due to the turbidity of mucin-supplemented media, 250-μl aliquots were taken every two hours for 12 h and then once more at 24 h after inoculation for each culture condition from cultures incubated at 37°C with shaking at 200 rpm and used to determine viable cells as CFU/ml. Each condition was assessed using three independent biological samples (*n* = 3).

### Quantification of metals with inductively coupled plasma-optical emission spectrometry (ICP-OES)

Autoclaved samples (1 ml) of SB and SB+M were lyophilized in 15-ml metal-free plastic conical tubes (VWR). Each dried sample was suspended in 0.25 ml of Barnstead-nanopure water and 1 ml of 50% metal-trace HNO_3_ was added into each tube. The samples were incubated at 60°C overnight before 3.75 ml of Barnstead-nanopure water were added to each digested sample, which was then filtered through a 0.22 μm nylon filter (Sterlitech). The samples were analyzed with a Perkin Elmer Optima 7000 DV ICP-OES system using 1% HNO_3_ as blank and the following plasma conditions: Argon to plasma flow rate, 15 l/min; nebulizer flow rate, 0.50 l/min; auxiliary flow rate, 0.20 l/min; power, 1,300 watts; plasma view, axial. The WinLab32 software package was used to run the samples and collect data. Standards containing 1.06 ppb, 10.7 ppb, 53.0 ppb and 106 ppb for each metal were used for calibration. Triplicate readings for each of three different SB and SB+M samples (*n* = 9) were used to quantify Cd^2+^, Co^2+^, Fe^3+^, Mg^2+^ and Zn^2+^ using the following wavelengths for each metal: 214.440, 236.830, 259.939, 280.271 and 213.857, respectively.

### Mucin degradation assays

Mucin degradation by bacterial cells and cell-free culture supernatants was tested using mucin that was biotin-labeled on either its carbohydrate or protein component following methods modified from Wiggins *et al*. [33]. Briefly, mucin was labeled with biotin on the carbohydrate or protein moiety using the HOOK Biotin Carbohydrate or HOOK Biotin Amine Reactive kits (GBiosciences), respectively, following the conditions suggested by the manufacturer. Carbohydrate biotin-labeled mucin was diluted to 2 μg/ml and protein biotin-labeled mucin was diluted to 0.5 μg/ml, and both stock samples were stored at -20°C prior to use [33]. Final concentrations of biotinylated mucin used in either the protein or carbohydrate degradation assays were obtained based on a validation assay to determine which concentrations of each type of labeled mucin gave relative luminescence units (RLUs) that were comparable to one another. To assess mucin degradation, 96-well white opaque plates were incubated at 4°C overnight with either carbohydrate- or protein-labeled mucin. The following day the plates were washed with sterile phosphate buffered saline (PBS) and inoculated with 50 μl of an overnight culture of ATCC 19606^T^ grown in SB and diluted to 10^6^ bacteria/ml or filter-sterilized culture supernatants. As a control, some wells were inoculated with sterile PBS. Plates were incubated for 24 h at 37°C, washed with PBS and then blocked overnight at 4°C with 5% bovine serum albumin dissolved in PBS. The plates were then washed with PBS and incubated with a 1:1,500 dilution of horseradish peroxidase (HRP)-streptavidin (Life Technologies) for 1 h at 4°C. After washing with PBS, the plates were developed using chemiluminescence. RLUs were determined using a FilterMax F5 multi-mode microplate reader (Beckman Coulter). Eight replicates of three independent biological samples for each experimental condition (*n* = 24) were used to validate the collected data.

### Biofilm quantification and scanning electron microscopy (SEM) analysis

One day prior to performing biofilm quantification assays, 12 x 75 mm plastic polystyrene tubes (Fisher) were coated with a sterile solution of 0.5% mucin dissolved in deionized H_2_O or washed with sterile deionized H_2_O alone. Tubes were stored at 4°C overnight. To commence the assay, tubes were washed twice with sterile deionized H_2_O and a 6-ml aliquot of SB broth was inoculated 1:100 with an overnight culture of ATCC 19606^T^ that was then dispensed as 1-ml aliquots into each tube. Tubes were incubated for 48 h at 37°C in a stationary incubator. The amount of biofilm formed on the walls of the tubes was determined using a crystal violet staining assay as previously described [34]. Quantity of biofilm was expressed as an OD_580_/OD_600_ ratio. Crystal violet assays were performed two times in triplicate for each experimental condition using fresh biological samples for each experiment (*n* = 6). To analyze biofilm structures using SEM, plastic coverslips were sterilized and coated with mucin overnight as described above and then prepared for SEM as previously reported [23]. SEM was performed on two biological replicates for each culture condition (*n* = 4).

### Transmission electron microscopy (TEM)

Bacteria were stab-inoculated into SA or SA containing 0.5% mucin (SA+M) plates and grown for 48 h at 37°C. Following incubation, a 5-μl drop of deionized H_2_O was added to the surface of the agar at the site of inoculation and a nitrocellulose-based, carbon coated, 300-mesh copper grid was immediately placed face down on the area covered by water for approximately 30 s. Grids were removed and 2 μl of 1.5% ammonium molybdate was added for 30 s. Excess stain was removed and the samples were air-dried. Imaging was performed on a JEOL JEM-1200 EX II transmission electron microscope at 120 keV. TEM was performed on two biological replicates for each culture condition (*n* = 4).

### Detection of bauA by western blotting

Bacteria were collected from 5-ml overnight cultures grown in SB or SB+M and cell pellets were resuspended in 1 ml of deionized H_2_O and sonicated on ice until clear. Whole cell lysates were adjusted to 1 mg/ml of protein [35]. The proteins present in each sample were analyzed by SDS-PAGE with 10% polyacrylamide gels using 10 μg of protein of each sample as described before [36]. Size-fractionated proteins were either detected by staining with Coomassie Brilliant Blue R or western blotting using anti-BauA polyclonal antibodies as previously described [37]. Immunocomplexes were detected by chemiluminescence using HRP-labeled protein A (Sigma-Aldrich). Total cell lysates of bacteria grown in LB broth or LB broth supplemented with 100 μM 2,2’-dipyridyl or 100 μM FeCl_3_ were included as controls for the detection of iron-regulated proteins [37]. Western blotting was performed three times using independent biological samples grown in each culture condition to confirm the presence or absence of BauA.

### Bacterial killing assays

These assays were performed using ATCC 19606^T^ and *E*. *coli* MG1655-Rif as predator and prey, respectively, as recently described [38]. The *E*. *coli* MG1655-Rif derivative was isolated by plating the wild type strain on LB agar containing increasing concentrations of rifampicin up to a final concentration of 100 μg/ml. *E*. *coli* MG1655 and MG1655-Rif SB cultures, and ATCC 19606^T^ SB or SB+M cultures were grown until they reached an OD_600_ of 0.5, washed and resuspended in 0.75 ml of saline solution. Equal volumes of prey and predator cultures were mixed and 10 μl were spotted on the surface of 0.22 μm filters placed on LB agar plates. After a 4-h incubation at 37°C, the filters were transferred to sterile 15-ml centrifuge tubes, bacteria were resuspended in 1 ml of SB and 10 μl of 10-fold serial dilutions were spotted on LB agar containing 100 μg/ml rifampicin. MG1655/MG1655-Rif mixtures were used as negative controls. Bacterial growth on the surface of the plates was recorded after incubation at 37°C for 36 h. Killing assays were repeated twice using independent biological samples each time.

### Polarization and infection of A549 human alveolar epithelial cells

A549 cells were polarized and infected as described before [39], using in this particular case 10^6^ bacteria/ml grown in either SB or SB+M and incubated for 48 h at 37°C in the presence of 5% CO_2_. In addition, polarized cells infected with bacterial cells grown in the presence of mucin were either infected and left untreated until the end of the experiment or treated with an additional 0.5% mucin in Hanks’ balanced salt solution without glucose (HBSSM) on the day of infection and every 24 h for the remainder of the infection by spotting 1 μl of HBSSM on the apical surface of the polarized A549 cells. The latter approach was used considering the observation that *A*. *baumannii* degrades mucin. Uninfected polarized cells treated with 0.5% mucin in HBSS every 24 h or left uninfected and untreated were used as negative controls. All polarized cell samples were fixed after 48 h of infection and analyzed by SEM as previously described [23]. Polarized cell assays were performed in triplicate for each condition using different biological samples each time.

### Cytotoxicity assays

A549 cells were seeded from a liquid-nitrogen frozen stock one week prior to the start of the assay and passaged in tissue culture flasks three times. Approximately 10^5^ cells/ml were then seeded into each well of a 96-well white opaque tissue culture plate and incubated for 24 h. The following day, the medium was aspirated from each well and the plate was washed with HBSS. Overnight bacterial cultures, grown in either SB or SB+M, were washed, suspended in HBSS to a density of 10^6^ bacteria/ml and then used to infect the A549 submerged monolayers, which were incubated for another 24 h. Sterile A549 cells incubated with HBSS or HBSSM were included as controls. To determine the cytotoxicity of each bacterial strain cultured with or without mucin, CellTiter-Glo luminescent cell viability assays (Promega) were performed twice in octuplet (*n* = 16) using fresh biological samples each time as previously described [40]. After incubation with each bacterial strain, the CellTiter plate was washed three times with fresh DMEM before the CellTiter-Glo reagent was added. Luminescence was then assessed using a FilterMax F5 multi-mode microplate reader (Beckman Coulter).

### Transcriptomic analyses

#### Growth conditions

Twenty-five ml of SB or SB+M were inoculated with 0.25 ml of overnight SB cultures in 125-ml flasks, which were incubated at 37°C with shaking at 200 rpm for 4 h or until an OD_600_ of approximately 0.5 was reached. Triplicates of three different biological replicates of bacteria cultured in SB in the absence or presence of 0.5% mucin were used to isolate total RNA as described below.

#### Library preparation and nucleotide sequencing

RNA was extracted from cells as described previously [41]. Briefly, 10 ml of culture were directly added to an equal volume of acid phenol (pH 4.3 ± 0.2) and 0.1% SDS followed by immediate incubation at 90°C for 10 min. The aqueous fraction was then extracted with acid phenol:chloroform (5:1, pH 4.5 ± 0.2) followed by chloroform. Total RNA was precipitated overnight at -20°C with 2.5 volumes of absolute ethanol. RNeasy on-column DNase I treatment was performed as *per* the manufacturer’s instructions (Qiagen). DNA removal was confirmed using QuantiTect SYBER Green PCR kit (Qiagen) with primers 3968 and 3969 (S1 Table) for the *recA* gene with amplification on a Bio-Rad CFX Connect real-time PCR detection system. RNA quantity was routinely estimated using a Nanodrop ND-2000 spectrophotometer and quality was assessed with an Agilent Bioanalyzer 2100 using the RNA 6000 Pico LabChip Kit (Agilent). DNA-free total RNA was rRNA depleted by treatment with the Ribo-Zero Magnetic Kit (Epicentre). Depletion was assessed using the RNA 6000 Pico LabChip Kit and the Bioanalyzer 2100. Cognate rRNA-depleted technical triplicates isolated from each of the three independent biological samples cultured in SB or SB+M were combined to prepare six (three for each culture condition) Illumina-compatible libraries using the NEBNext Ultra Directional RNA Library preparation kit (New England Biolabs Inc.) according to the manufacturer’s instructions. All purifications were performed with Agencourt AMPure XP Beads (Beckman Coulter, Inc.) as described in the NEBNext protocol. After purification, the size of the DNA amplicons was assessed with the Agilent High Sensitivity DNA Kit on a Bioanalyzer 2100. The KAPA Library Quantification kit (Kapa Biosystems Inc.) was used to quantify libraries on a BioRad CFX Connect real-time PCR detection system. MiSeq reagent v.3 was used for sequencing on a MiSeq platform (Illumina). Demultiplexing and sequence alignment to the *A*. *baumannii* ATCC 17978 complete genome, including the plasmids pAB1 and pAB2 [42], was done with the Illumina software and the 7.0.4 version of the CLC Genomics Workbench software package. The nucleotide sequence of differentially transcribed genes was further analyzed using Blast2GO 4.0.7 (Young 2005) and the KEGG metabolic pathway mapping system.

#### Real-time RT-PCR

The differential expression of a select set of genes was determined by real-time RT-PCR (qRT-PCR) using as templates the RNA obtained for the RNA-Seq approach described above and the primers listed in S1 Table. cDNA libraries were constructed using the iScript cDNA synthesis kit (Bio-Rad Laboratories) from 10 ng of total RNA according to the manufacturer’s protocol. Transcript levels were then analyzed using iQ SYBR Green (Bio-Rad Laboratories) following the manufacturer’s recommendations. As an internal control, primers 3968 and 3969 (S1 Table) were used to amplify a 160-bp fragment of the *recA* gene. To assess type I pili gene transcription, primers 3204 and 3205 (S1 Table) were used to amplify a 165-bp fragment of *csuA/B*. To assess the transcription of the *bauA* gene coding for the iron-regulated acinetobactin receptor protein, primers 3970 and 3971 (S1 Table) were used to amplify a 156-bp fragment of this gene. To assess transcription of the predicted benzoate degradation operon, primers 4402 and 4403 (S1 Table) were used to amplify a 215-bp fragment of the HMPREF0010_RS07075 ATCC 19606^T^ gene coding for a *benP* ortholog. A 178-bp fragment of A1S_1307 (ATCC 19606^T^ gene HMPREF0010_RS10250), which is predicted to code for a TssH ATPase ortholog, was used to assess the differential expression of T6SS genes using primers 4404 and 4405 (S1 Table). Finally, primers 4406 and 4407 (S1 Table) were used to amplify a 162-bp fragment of the A1S_1343 gene (ATCC 19606^T^ gene HMPREF0010_RS10070), which codes for a *paaH* ortholog potentially involved in phenylacetic acid metabolism. The following cycling conditions for qRT-PCR were used on a Bio-Rad CFX Connect real-time PCR detection system: 95°C for 3 min followed by 40 cycles of 95°C for 3 s and 60°C for 45 s. Analysis of relative expression data was performed in triplicate on three biological replicate samples cultured in SB or SB+M. Relative expression of *csuAB*, *bauA*, A1S_1209 (*benP*), A1S_1307 (*tssH*) and A1S_1343 (*paaH*) were quantified by the standard curve method in which serial dilutions of cDNA samples served as standards. The expression of each of these genes was normalized to the expression of *recA*, which was not significantly affected by the presence or absence of mucin in the medium according to RNA-Seq data. Amplification reactions containing no cDNA template were used as negative controls. All qRT-PCR experiments were done in triplicate using three independent biological samples for each gene and condition (*n* = 9).

### Statistics

Statistical analyses provided as part of the GraphPad InStat software package (GraphPad Software, Inc.) were used to analyze the statistical significance of data sets. One-tailed Student’s *t-*tests were used to analyze the significance of biofilm data, while qRT-PCR, mucin-labeled degradation and cytotoxicity results were analyzed using one-tailed Mann-Whitney tests. Growth curve data were analyzed for statistical significance by comparing CFUs/ml using the Kruskal-Wallis test with the Dunn’s multiple comparisons post-hoc test. *P* values ≤ 0.05 were considered statistically significant for all aforementioned assays. Error bars represent the standard error for each data set shown in the figures.

### Accession number(s)

The RNA-Seq data generated as a result of this work have been approved by NCBI and assigned the GEO accession number 100582.

## Results

### The presence of mucin affects *A*. *baumannii*-host cell interactions and cytotoxicity

**Fig 1:**
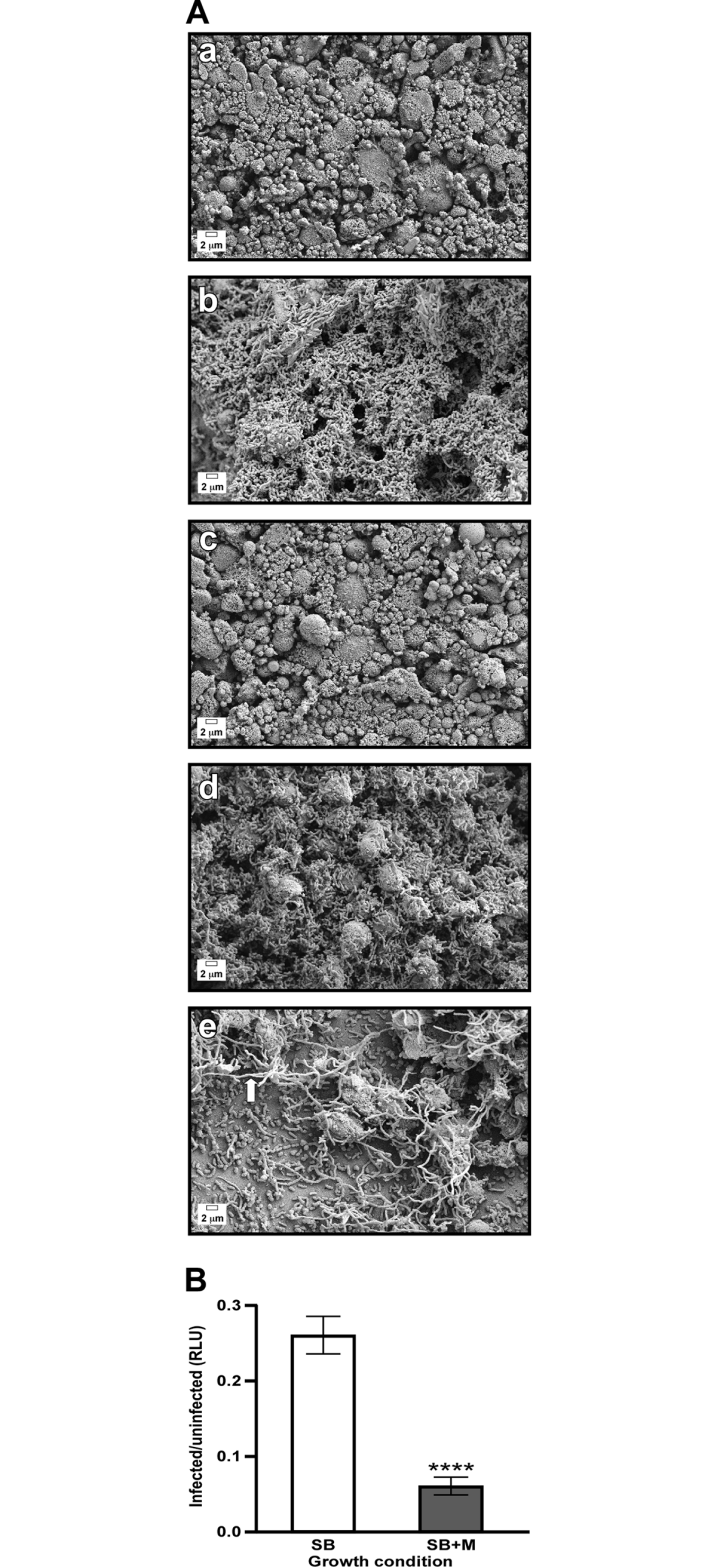
Effect of mucin on the interaction of *A*. *baumannii* with human alveolar epithelial cells and virulence. (A) Bacteria-alveolar epithelial cell interactions. SEM of sterile polarized A549 alveolar epithelial cells cultured in the absence (a) or presence (c) of exogenous mucin. Micrographs b and d correspond to A549 polarized cells infected with ATCC 19606^T^ bacteria cultured in SB or SB+M prior to infection, respectively. Micrograph e corresponds to A549 polarized cells infected in the presence of exogenous mucin with ATCC 19696^T^ bacteria cultured in SB+M prior to infection. The white arrow indicates a filamentous bacterial cell. Scale bars, 2 μm. Micrographs were taken at a magnification of 5,000x. (B) Bacterial cytotoxicity against A549 submerged monolayers. Relative luminescence units (RLUs) represent the relative amount of ATP detected with intact A549 cells that remained after incubation with bacteria cultured in SB (white bar) or SB+M (grey bar) prior to infection in relation to the amount of ATP detected with uninfected epithelial cells. Cytotoxicity assays were performed using 16 replicates. Statistically different values (*P* ≤ 0.0001) are identified by four asterisks and error bars represent the standard error of each data set.

To gain a better understanding of the previously described interaction between *A*. *baumannii* ATCC 19606^T^ and biotic environments [22, 23], polarized A549 cells were infected with ATCC 19606^T^. Polarized A549 human alveolar epithelial cells were chosen as an *ex vivo* virulence model because they mimic the host lung environment. As part of the polarization process, A549 cells differentiate to form tight junctions, secrete surfactant via lamellar bodies and form structures that resemble those formed in the host [27, 43]. Infection with ATCC 19606^T^ bacteria, previously cultured in SB, resulted in dense biofilms on top of polarized cells, as well as significant damage to the A549 cells and the surfactant layer when compared to controls (Fig 1A, compare panels a and b). Based on these observations, we hypothesized that ATCC 19606^T^ senses and responds to a component of either the A549 cells or the surfactant being secreted by them. Since the primary biological component secreted in large quantities by A549 cells is mucin [24–26], we tested the effect of the presence of exogenous mucin. For this purpose we adapted protocols used for the study of *P*. *aeruginosa*, another relevant human respiratory pathogen, that are based on the addition of commercially available PGM to culture media based on the observation that MUC5AC is the major component of gastric and respiratory mucins [19, 44–47]. Mucin was added to SB or SA, a low nutrient media, to a final concentration of 0.5%, which is within the range usually found in respiratory secretions [21]. Mucin was added to the media to grow bacteria prior infection or added to the tissue culture media for the duration of the infection of A549 polarized cells. Infection of A549 cells with bacteria cultured in SB+M before inoculation resulted in a significant increase in cell damage with extensive degradation of the surfactant layer when compared to samples infected with bacteria cultured in SB (Fig 1A, compare panels b and d). The cytotoxicity and surfactant damage was further enhanced when 0.5% mucin was present in the culture of the bacterial inocula as well as throughout the duration of the infection. Under this condition, ATCC 19606^T^ bacteria destroyed most of the surfactant and polarized cell layers leaving few A549 cells intact, with bacteria forming biofilms on top of the trans-well membrane (Fig 1A, panel e). In addition, long, filamentous bacteria (Fig 1A, panel e white arrow) were abundant in this growth condition, but were not detected in the other tested conditions (Fig 1A, panels b and d). The addition of exogenous mucin to sterile polarized cells did not cause any apparent damage or change to the cell layers when compared to untreated, sterile polarized A549 cells (Fig 1A, panels a and c). These observations are consistent with the effect mucin has on ATCC 19606^T^ bacteria when co-cultured with submerged A549 monolayers. Whereas ATCC 19606^T^ bacteria cultured in SB produce detectable cytolytic activity, cytolysis was drastically increased when the A549 monolayers were infected with bacteria cultured in SB+M as reflected by a significant (*P* < 0.0001) reduction in luminescence (Fig 1B). The incubation of sterile A549 monolayers in the presence or absence of mucin did not cause significant changes in cytolytic activity (data not shown). All these results indicate that culturing *A*. *baumannii* in the presence of mucin significantly enhances bacterial cytolytic activity as determined by two different *ex vivo* experimental approaches.

Taken together, these observations demonstrate that the presence of mucin either in the culture medium used to prepare the inocula or during the infection of human epithelial cells, while not causing detectable effects on human epithelial cells, significantly affects the interaction of *A*. *baumannii* with human host cells and significantly increases its cytotoxic activity.

### Mucin induces a global differential transcriptional response in *A*. *baumannii*

We predicted that the significant effects of mucin on host-pathogen interactions and cytotoxicity responses described above involved the coordinated expression of a large number of genes. Therefore, we used RNA-Seq to examine the global transcriptional response of ATCC 19606^T^ to the presence of mucin in a nutrient limited medium such as SB. Sequencing of cDNA libraries prepared using total RNA isolated from bacteria grown in SB or SB+M generated an average of 44,633,336 and 48,320,630 total reads, respectively, for the three replicates for each experimental condition, with an overall average length of 74 nucleotides for all six sequenced libraries (S2 Table). An average of 97.27% and 98.83% of the reads obtained from bacteria cultured in SB and SB+M, respectively, were mapped to the genome of *A*. *baumannii* ATCC 17978 [42], using the CLC Genomics Workbench software package, since a closed fully annotated ATCC 19606^T^ genome is not currently available (S2 Table). Additional information on the quality of the RNA-Seq data is provided in S2 Table as well as S1 Fig, which shows that intra- and inter-group expression means are comparable with similar overall variations in expression among the libraries prepared using RNA isolated from bacteria cultured in SB or SB+M. Multidimensional scaling (MDS) analysis showed that there is a significant difference between the data collected from cells cultured in SB and SB+M, with cognate data grouping together as shown in S2 Fig. The overall transcriptional response of ATCC 19606^T^ cells to the presence of mucin in the culture medium is represented by the volcano plot displayed in S3 Fig. This figure shows that the presence of mucin induced a global differential transcriptional response, which encompasses a total of 427 predicted protein-coding genes that were differentially transcribed, including 232 and 195 transcriptional units up- and down-regulated, respectively (S3 and S4 Tables), when the data were analyzed using a fold-change threshold of 2 and statistically significant difference values (*P* ≤ 0.05). Overall, the presence of mucin in the culture medium promoted the expression of genes coding mainly for cellular and metabolic processes as well as single-organism processes (S4A Fig). Gene ontology (GO) analysis of the differentially transcribed genes showed that the addition of mucin to SB affects a wide range of cellular processes, including oxidation-reduction, electron carrier/transport and cellular and macromolecular metabolic processes, as well as porin and channel activities and genes coding for benzoate and 3,4-dihydroxybenzoate metabolism (S4B Fig). Taken together, these results indicate that the presence of mucin in the extracellular environment affects the overall physiology of *A*. *baumannii*. This effect could reflect the metabolic versatility and adaptability of this pathogen that successfully persists in different ecological niches including those found in the human host.

### *A*. *baumannii* degrades and utilizes mucin as an energy and nutrient source

**Fig 2:**
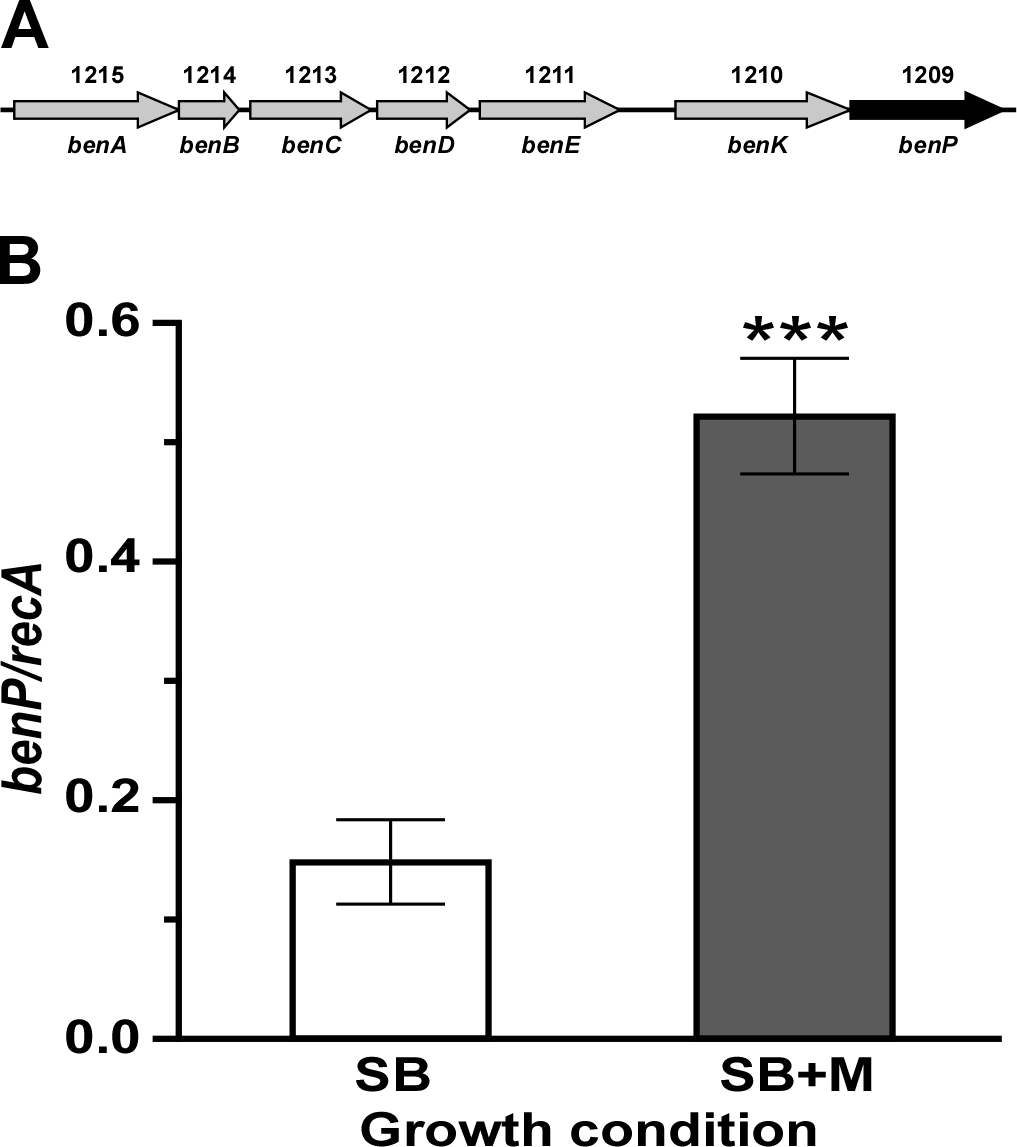
Differential transcription of genes coding for benzoate transport and metabolism functions in response to the presence of mucin. (A) A1S_1215-A1S_1206 genomic region coding for BenA/B/C/D/E/K/P orthologs. The arrows represent each coding region and its direction of transcription. The grey arrows represent genes up-regulated in response to the presence of mucin. The black arrow indicates the gene (*benP*, A1S_1209) that was used to confirm the mucin-mediated up-regulation effect by qRT-PCR. Numbers above the arrows represent cognate A1S_ gene annotation numbers, and gene names are indicated below each arrow. (B) qRT-PCR analysis of the differential expression of A1S_1209 in bacterial cells cultured in SB (white bar) or SB+M (grey bar) performed using nine replicates. Significantly different values (*P* ≤ 0.001) are identified by three asterisks and error bars represent the standard error of each data set.

The presence of mucin significantly up-regulates the transcription of genes coding for metabolic functions, with the 28 genes coding for predicted functions involved in benzoate/catechol transport and catabolism being the largest group of genes differentially transcribed in response to mucin (Table 1). This group includes 19 predicted coding regions clustered in three different genomic locations representing potential polycistronic operons; A1S_1209-A1S_1215, A1S_1843-A1S_1850 and A1S_1884-A1S_1888 (Table 1). It is important to note that some coding regions may represent a single gene due to potential sequencing errors associated with the annotation of the ATCC 17978 genome (Table 1, genes labeled with an asterisk). Genes coding for predicted Ben and Cat/Pca proteins involved in the transport and catabolism of aromatic compounds via the catechol branch of the β-ketoadipate pathway, which have been identified and analyzed in more detail in the *A*. *baylyi* ADP1 environmental isolate [48, 49], are included in this set of mucin-induced genes (Table 1). qRT-PCR analysis of A1S_1209, the last component of the potential A1S_1209-A1S_1215 7-gene polycistronic operon that codes for the predicted benzoate transport protein BenP (Fig 2A), validated the significant (*P* = 0.0002) transcriptional up-regulation of this gene and operon in response to the presence of mucin in the culture medium (Fig 2B). Overall, these observations not only indicate the remarkable and interesting effect of mucin on the expression of catechol/benzoate transport and utilization functions, but also serve as a guide to further explore the role of these functions in the pathophysiology of *A*. *baumannii*. This goal could be achieved by generating and testing proper isogenic derivatives affected in the expression of mucin-regulated catechol/benzoate transport and utilization functions without negative effects on central metabolic pathways.

**Table 1:** *A*. *baumannii* ATCC 19606^T^ metabolic genes with increased expression in the presence of mucin.

Gene Identifier^#^	Fold Change	*P* value	Predicted function
***Benzoate/catechol transport and catabolism***			
A1S_0954	3.675535189	5.93865E^-05^	Alpha/beta hydrolase family protein, CatD
**A1S_1209**	**5.473142215**	**0.002552661**	**Benzoate transport porin, BenP**
A1S_1210	5.025999281	0.006720615	Major facilitator superfamily transporter, BenK
A1S_1211	4.50743886	8.89275E^-06^	Benzoate transporter, BenE
benD (A1S_1212)^§^	7.230360928	0.000872548	1,6-dihydroxycyclohexa-2,4-diene-1-carboxylate dehydrogenase, BenD
A1S_1213	6.923948731	0.003951293	Benzoate 1,2-dioxygenase electron transfer component, BenC
A1S_1214	8.411393456	0.002683066	Benzoate 1,2-dioxygenase subunit beta, BenB
A1S_1215	7.426423484	0.00201372	Benzoate 1,2 dioxygenase subunit alpha, BenA
A1S_1843	2.800652852	0.024512529	Muconate cycloisomerase I, benzoate degradation, CatB
A1S_1844	4.792432953	0.001106926	Muconolactone Delta-isomerase, CatC
A1S_1845	4.711557575	0.001143271	Catechol 1,2-dioxygenase, CatA
A1S_1846	2.700134212	0.000836053	3-oxoadipate CoA-transferase subunit A, CatI/PacI
A1S_1847	5.388171769	0.000601207	3-oxoadipate CoA-transferase subunit B, CatJ/PcaJ
A1S_1849	4.014897458	0.000112128	Beta-ketoadipyl CoA thiolase, CatF/PcaF
A1S_1850	3.251441888	0.005491257	3-oxoadipate enol-lactonase 2, CatD
A1S_1859	7.579664989	0.003910183	Aromatic-ring-hydroxylating dioxygenase small subunit
A1S_1860*	8.113167493	0.004013873	Aromatic-ring-hydroxylating dioxygenase large subunit, Rieske (2Fe-2S) protein
A1S_1861*	7.792995922	0.006111932	Aromatic-ring-hydroxylating dioxygenase large subunit, Rieske (2Fe-2S) protein
A1S_1868*	4.925893295	0.002549497	Aromatic compound porin protein
A1S_1869*	4.785364141	0.009283729	Aromatic compound porin protein
A1S_1884	2.672152015	1.04452E^-05^	Protocatechuate 3,4-dioxygenase subunit alpha, PcaG
A1S_1885	2.234148816	2.88521E^-05^	Protocatechuate 3,4-dioxygenase subunit beta, PcaH
A1S_1886	2.556497961	2.75334E^-07^	Gamma-carboxymuconolactone decarboxylase, PcaC
A1S_1887*	2.275183296	8.0351E^-06^	4-hydroxybenzoate transporter, PcaK
A1S_1888*	2.577267732	6.89506E^-07^	4-hydroxybenzoate transporter, PcaK
A1S_1891	3.816740734	0.000529673	Beta-ketoadipyl CoA thiolase, PcaF
A1S_1892	5.012024813	0.000100525	Beta-ketoadipyl CoA thiolase, PcaF
A1S_1894	2.946424012	0.008872698	3-oxoadipate CoA-transferase subunit A, PcaI
***Phenylacetate catabolism***			
A1S_1341	7.870503414	0.042039166	Enoyl-CoA hydratase, PaaF
A1S_1342	8.861252528	0.039866679	Epoxyphenylacetyl-CoA isomerase, PaaG
**A1S_1343**	**10.85004468**	**0.023505204**	**3-hydroxyacyl-CoA dehydrogenase, PaaH**
A1S_1344	13.15884402	0.005538184	Beta-ketoadipyl CoA thiolase, PaaJ
A1S_1345*	15.00769651	0.001207025	Phenylacetate-CoA ligase, PaaK
A1S_1346*	14.78059953	0.00014185	Phenylacetate-CoA ligase, PaaK
A1S_1347	8.214779513	0.00066628	Phenylacetic acid degradation operon repressor protein, PaaX
A1S_1348	10.13187857	3.69166E^-05^	Phenylacetic acid degradation protein, PaaY
A1S_1349	5.64077843	5.33322E^-05^	Thioesterase domain-containing protein, PaaI
A1S_1856	9.373208823	0.000696445	*p*-hydroxyphenylacetate hydroxylase C1:reductase component
***Electron trasport*, *energy production***			
A1S_0005	4.876856033	5.79461E^-07^	Cytochrome b precursor
A1S_0076	2.058803438	0.009488764	Aconitate hydratase
A1S_0754	2.187429899	0.003373818	NADH:ubiquinone oxidoreductase subunit C/D
A1S_0755	2.307700992	0.001117444	NADH dehydrogenase I E subunit E
A1S_0756	2.469437059	0.000653644	NADH dehydrogenase I subunit F
A1S_0757	2.366086417	0.002066849	NADH dehydrogenase subunit G
A1S_0758	2.287136558	0.003517374	NADH dehydrogenase subunit H
A1S_0759	2.275271124	0.003174267	NADH dehydrogenase subunit I
A1S_0761	2.403445757	0.001435073	NADH dehydrogenase I subunit K
A1S_1924	6.429886857	1.88258E^-06^	Cytochrome d terminal oxidase polypeptide subunit I, CydA
A1S_1925	8.114881419	2.60992E^-06^	Cytochrome d terminal oxidase polypeptide subunit II, CydB
A1S_2166	2.026413775	0.018869709	Cytochrome bo(3) ubiquinol oxidase, CyoA
A1S_2338	2.321699725	0.000396794	Malate dehydrogenase, MaeB
A1S_2475	2.285469911	0.002414222	Isocitrate dehydrogenase (Icd)
sucA (A1S_2715)	2.048840719	0.007128676	2-oxoglutarate dehydrogenase
A1S_2716	2.19742636	0.003297483	2-oxoglutarate dehydrogenase, SucB
A1S_2717	2.23412058	0.002492584	Dihydrolipoamide dehydrogenase
A1S_2718	2.207779293	0.002958372	Succinyl-CoA synthetase subunit beta, SucC
A1S_2719	2.16669819	0.003352092	Succinyl-CoA synthetase subunit alpha, SucD
***Amino acid catabolism*, *peptidases and proteases***			
A1S_0956	2.177325999	0.014618736	L-aspartate dehydrogenase
A1S_0957	2.43728155	0.007415014	Betaine-aldehyde dehydrogenase
A1S_0958	2.423328781	0.004294133	Acetolactate synthase large subunit
A1S_1372	3.802051601	0.000345683	Hydroxymethylglutaryl-CoA lyase, MvaB
A1S_1373	3.25732149	0.003228408	3-methylcrotonyl-CoA carboxylase subunit alpha
A1S_1374	3.345393954	0.002883219	3-methylglutaconyl-CoA hydratase
A1S_1375	3.012793835	0.01043401	3-methylcrotonyl-CoA carboxylase subunit beta
A1S_1376	3.238990899	0.004131094	Isovaleryl-CoA dehydrogenase
A1S_1610	2.081951258	2.45929E^-05^	Zn-dependent metalloendopeptidase
A1S_1852	4.637517532	0.001480446	Phenylacetaldehyde dehydrogenase, FeaB
A1S_1853	4.401870192	0.007821506	Putative tynE
tynA (A1S_1854)	4.69193766	0.009162041	Tyramine oxidase, copper-requiring
A1S_2232	2.778516617	5.45342E^-10^	Methylmalonate-semialdehyde dehydrogenase (MmsA)
A1S_2589	4.081294884	4.20755E^-06^	Aminoacylase-2/carboxypeptidase-Z family hydrolase, AbgB
clpX (A1S_0477)	2.049805338	0.00307486	ATPase and specificity subunit of ClpX-ClpP ATP-dependent serine protease
***Amino acid/peptide transport***			
A1S_1467	2.003213585	0.023546957	Glutamate symport transmembrane protein, GltT/GltP
A1S_1490	2.962050012	0.001116133	Glutamate/aspartate periplasmic-binding protein, GltI
A1S_1491	3.600540965	5.80563E^-05^	Glutamate/aspartate transport permease protein, GltJ
A1S_1492	3.366102462	0.000230756	Glutamate/aspartate transport permease protein, GltK
A1S_1493	3.441857707	9.51136E^-05^	Glutamate/aspartate transport ATP-binding protein, GltL
A1S_1956	3.473051794	0.000481621	Amino acid permease, AnsP
A1S_2449	5.39149804	0.003395911	Aromatic amino acid APC transporter, AroP
A1S_2538	2.091909892	0.011128303	Outer membrane protein CarO precursor
A1S_2633	2.703468173	0.000182712	D-alanine/D-serine/glycine transport protein
A1S_2763	2.206786121	0.000130274	Aromatic amino acid APC transporter
***Lipid catabolism and transport***			
prpB (A1S_0073)	2.256823408	0.001232441	2-methylisocitrate lyase—PrpB
A1S_0103	2.291988117	0.016345817	3-hydroxyisobutyrate dehydrogenase
A1S_0104	2.214320009	0.031303506	Acetyl-CoA synthetase/AMP-(fatty) acid ligase—AcsA
A1S_0105	2.217156233	0.01599743	Acyl-CoA dehydrogenase
A1S_0106	2.557525827	0.00316474	Anoyl-CoA hydratase/isomerase
A1S_0107	2.960278778	0.001415848	Anoyl-CoA hydratase
A1S_0108	3.385075781	0.000404392	Major facilitator superfamily metabolite/H(+) symporter
fadA (A1S_0305)	2.153099142	0.006087876	3-ketoacyl-CoA thiolase, FadA
A1S_0591	2.126336588	0.000478223	Long-chain-fatty-acid-CoA ligase
A1S_1261	2.350407725	1.15161E^-08^	3-hydroxyacyl-CoA dehydrogenase
A1S_1378	5.408398444	0.000219374	Long chain fatty-acid CoA ligase
A1S_1379	3.767670502	0.000390434	SAM-dependent methyltransferase
A1S_1380	5.770084898	1.78561E^-06^	DcaP-like porin protein
A1S_1381	2.711658853	2.33232E^-07^	GDSL-like Lipase
A1S_1814	2.401041687	9.93427E^-06^	Bile acid:sodium symporter/arsenical resistance protein
A1S_1815	3.032423133	1.96739E^-07^	Long-chain fatty acid-CoA ligase
A1S_1816	2.092615938	0.00042146	Long-chain fatty acid transport protein
A1S_1817	3.361943473	5.24771E^-12^	Acyl-CoA dehydrogenase
A1S_1818	3.216329497	6.00059E^-09^	MaoC-like dehydratase
A1S_1819	2.906607377	4.13969E^-10^	3-hydroxyacyl-CoA dehydrogenase
A1S_1821	2.804324541	5.17674E^-09^	Short chain dehydrogenase
A1S_1858	7.366319481	0.004179325	Short-chain dehydrogenase
A1S_2308	2.183985812	3.11462E^-06^	Triacylglycerol lipase
A1S_2348	3.192409387	2.16553E^-10^	Triglyceride lipase
A1S_2702	2.830055349	3.73942E^-08^	1,3-propanediol dehydrogenase
A1S_2773	2.336819778	0.004592853	Long-chain fatty acid transport protein
A1S_3160	4.406299142	2.84633E^-06^	Lipase

^#^Genes grouped together represent gene clusters transcribed in the same direction, some of which are potential polycistronic operons, and predicted to code for related functions.

^§^A1S_numbers represent cognate gene identifiers if these were annotated following the system used to annotate most of the predicted genomic coding regions.

*Gene identifiers that could represent a potential single coding region due to DNA sequencing errors.

Bolded gene identifiers, differential expression tested by qRT-PCR.

**Fig 3:**
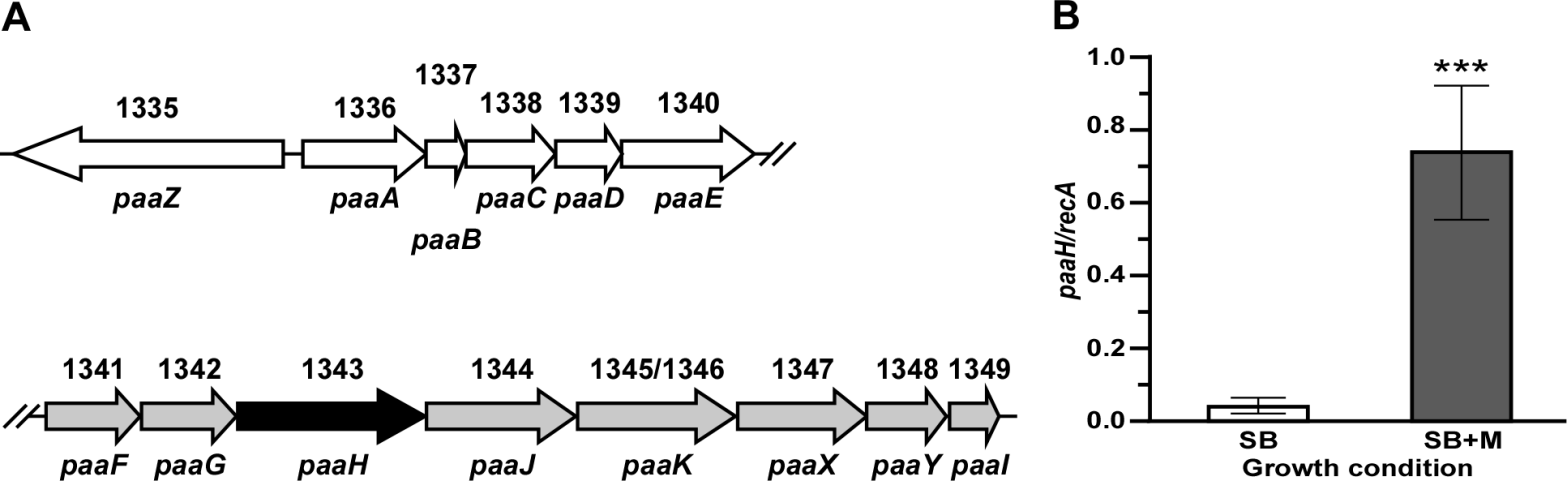
Differential transcription of the *paa* operon in response to the presence of mucin. (A) ATCC 19606^T^ genomic cluster coding for phenylacetate degradation functions. The arrows represent each coding region and its direction of transcription. The white and grey arrows represent genes constitutively and differentially expressed in response to the presence of mucin, respectively. The black arrow indicates the gene (*paaH*, A1S_1343) that was used to confirm the mucin-mediated up-regulation effect by qRT-PCR. Numbers above the arrows represent cognate A1S_ gene annotation numbers, and gene names are indicated below each arrow. Coding regions A1S_1345 and A1S_1346 are represented as a single coding region since potential nucleotide sequencing errors could have resulted in the annotation of two open reading frames. (B) qRT-PCR analysis of the differential expression of A1S_1343 in bacterial cells cultured in SB (white bar) or SB+M (grey bar) performed using nine replicates. Significantly different values (*P* ≤ 0.001) are identified by three asterisks and error bars represent the standard error of each data set.

The A1S_1345 and A1S_1346 genes, which most likely represent a single coding region due to sequencing errors, together with A1S_1344, all of which code for enzymes involved in phenylacetate catabolism, represent the highest induced predicted genes with more than 10-fold changes in gene expression in response to the presence of mucin (Table 1). These three genes are part of the mucin-induced 8-gene cluster A1S_1341-A1S_1349, which maps within the 13-gene *paa* operon (Fig 3A). This operon includes genes coding for enzymatic activities as well as the PaaX repressor (A1S_1347), which together with the product of *paaY* (A1S_1348) control the expression of all *paa* genes in a phenylacetic acid-CoA dependent manner [50]. qRT-PCR analysis of the A1S_1343 (*paaH*) coding region using RNA samples collected from bacteria cultured in SB or SB+M not only confirmed the significant (*P* = 0.0003) up-regulation of this gene in response to the presence of this glycoprotein, but also showed that its expression is barely detectable in cells cultured in the absence of mucin (Fig 3B). Mucin also up-regulates the expression of A1S_1856 (Table 1), which is involved in the transformation of 4-hydroxyphenylacetate into pyruvate and succinate semialdehyde by *A*. *baumannii* [51].

**Fig 4:**
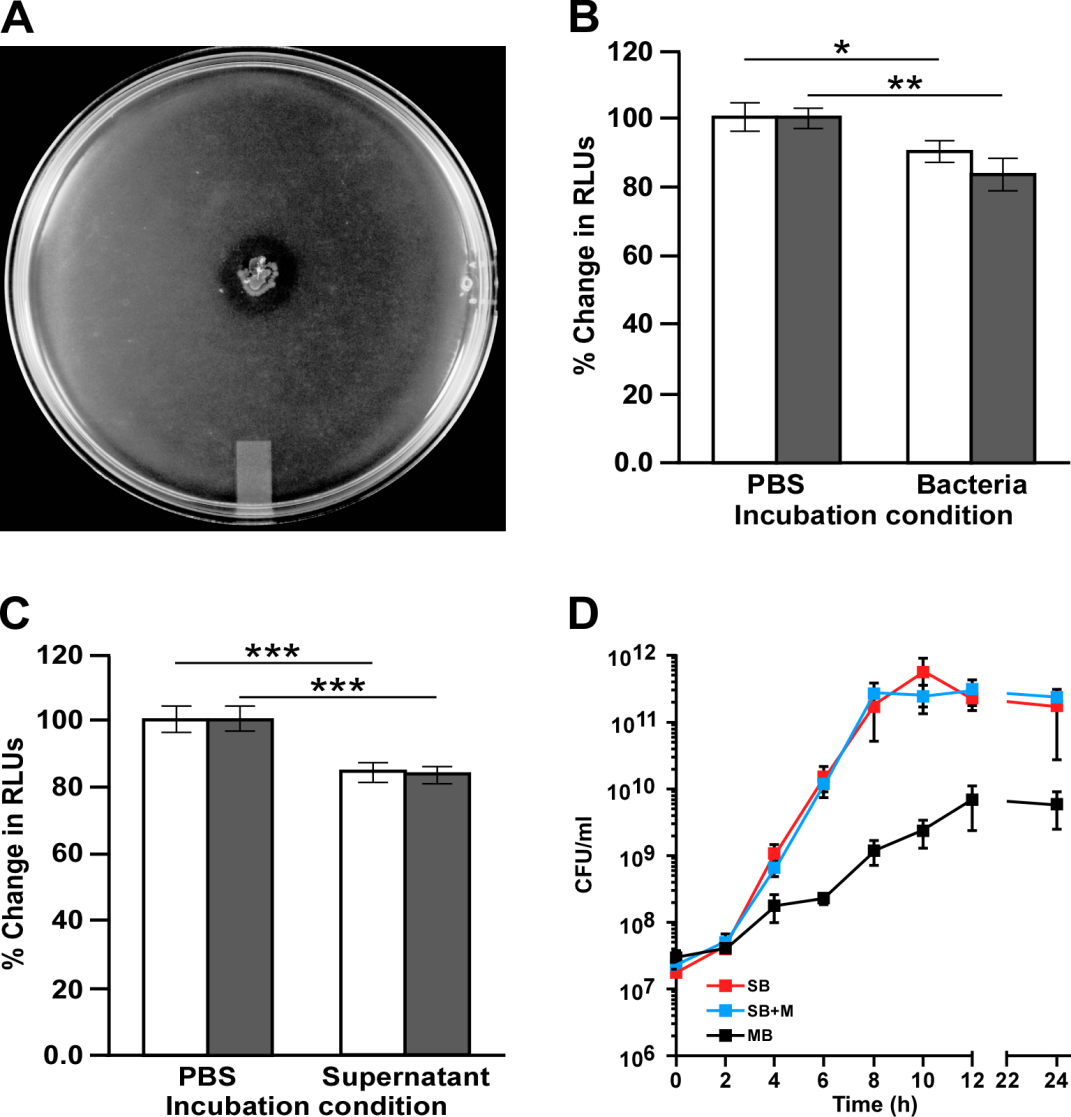
Utilization and degradation of mucin by *A*. *baumannii*. (A) Image of ATCC 19606^T^ cells grown on the surface of a SA plate supplemented with 0.5% mucin for 24 h at 37°C. The halo surrounding the colony represents mucin degradation. Representative image of experiments performed three times using three technical and fresh biological samples for each experiment (*n* = 9). Degradation of carbohydrate- (white bars) and protein-labeled mucin (grey bars) by ATCC 19606^T^ bacterial cells (B) or cell-free culture supernatants (C) performed using eight technical replicates for each condition. Results are displayed as a percentage of the negative control RLUs. Significantly different values are identified by one, two, and three asterisk representing *P* values ≤ 0.05, ≤ 0.01 and ≤ 0.001, respectively. Error bars represent the standard error of each data set. (D) Growth curves of ATCC 19606^T^ cells cultured in SB, SB+M or MB for 24 h at 37°C performed using three independent biological samples for each culture condition (*n* = 9). Error bars represent the standard error of each data set.

The up-regulation of all genes described above, which code for converging catabolic functions critical for the utilization of aromatic compounds as nutrient and energy sources, match the increased transcription of 19 genes coding for critical cellular functions associated with Krebs cycle and electron transport processes required for energy production (Table 1). This observation suggests that *A*. *baumannii* could degrade mucin to use it as a nutrient source. Such a possibility is supported by the observation that growth of ATCC 19606^T^ cells on the surface of SA supplemented with 0.5% mucin results in increasing transparency of the agar surrounding the bacterial colony (Fig 4A). The potential ability of *A*. *baumannii* ATCC 19606^T^ to degrade mucin was further confirmed with mucin degradation assays using as a substrate mucin biotinylated in either the protein or carbohydrate moiety. Fig 4B shows that the presence of bacteria in the reaction mixture produced a 10.01 ± 2.92% and 16.77 ± 3.68% reduction in the luminescence of mucin labeled at the carbohydrate or protein moieties, respectively, a significant difference (*P* values 0.0201 and 0.0036, respectively) when compared with the signals produced by cognate labeled samples incubated with sterile PBS under the same experimental conditions. Incubation of either carbohydrate- or protein-labeled mucin with ATCC 19606^T^ cell-free culture supernatants also caused a significant signal reduction (*P* values 0.0007 and 0.0005, respectively) with a 15.69 ± 2.06% and 16.52 ± 2.48% decrease in luminescence, respectively (Fig 4C).

Taken together, the results described above suggest that *A*. *baumannii* ATCC 19606^T^ possesses an uncharacterized mucinase activity, some of which is secreted into the culture supernatant and allows this pathogen to degrade and use an important host glycoprotein as an energy and nutrient source. This possibility is supported by the mucin-mediated up-regulated expression of 15 genes coding for predicted proteases, peptidases and amino acid catabolic enzymes, as well as the enhanced transcription of 10 amino acid and peptide permeases and transport proteins (Table 1). Furthermore, the presence of mucin in the culture medium induced the transcription of 27 genes predicted to code for proteins involved in lipid metabolism and transport, which represent the second largest set of mucin-induced genes after those involved in benzoate/catechol transport and catabolism (Table 1).

The possibility that the presence of mucin induces the production of lipid degradation products is particularly supported by the significantly increased expression of the A1S_0073 and A1S_0104 genes when bacteria were cultured in SB+M (Table 1). A1S_0073 codes for a predicted PrpB 2-methylisocitrate lyase ortholog, a component of the methylcitrate cycle involved in the α-oxidation of the short chain fatty acid (SCFA) propionate to pyruvate in aerobic bacteria including *Escherichia coli* [52, 53] and *Salmonella typhimurium* LT2 [54]. This metabolic process starts with the activation of propionate to propionyl-CoA that is then condensed with oxaloacetate to produce methylcitrate. This product is dehydrated to 2-methyl-*cis*-aconitate and then re-hydrated to produce 2-methyl-isocitrate, which is finally cleaved into pyruvate and succinate by the action of PrpB [54]. A1S_0104 codes for a predicted AcsA acetyl-CoA synthetase/AMP-(fatty) acid ligase, an enzyme produced by different bacteria to convert the SCFA acetate into acetyl-coenzyme A (Ac-CoA), which is then used for energy production and biosynthesis processes. This enzyme, also known as AMP-forming Ac-CoA synthetase because of the formation of an acyl-AMP intermediate that is then transformed into Ac-CoA by acyl-CoA synthetase, is a critical component of the high-affinity pathway cells use when the acetate concentration is low [55]. The potential ability of ATCC 19606^T^ to catabolize propionate and acetate using PrpB and AcsA activities when cultured in the presence of PGM is further supported by the observation that these two enzymes are critical for the degradation of these two SCFAs when *Pseudomonas aeruginosa* is cultured in a medium containing PGM and inoculated with PGM-degrading fermenting anaerobic bacteria [56].

The observation that mucin could serve as a nutrient source prompted us to determine if mucin affects the growth of ATCC 19606^T^ cells when cultured in SB at 37°C in a shaking incubator. This analysis showed that although the presence of mucin enhanced the transcription of genes coding for energy production and metabolic processes, it does not result in significant differences in CFUs/ml detected in samples collected from cultures incubated in SB or SB+M over the course of 24 h (Fig 4D). This approach also showed that mucin broth (MB), 0.5% mucin suspended in deionized water, was capable of promoting bacterial growth more than 100-fold after 24 h incubation at 37°C in a shaking incubator, although to levels lower than those achieved using SB or SB+M under the same culture conditions. Interestingly, this growth response is similar to that displayed by *P*. *aeruginosa* PA14, which showed reduced growth in a chemically defined medium containing 3% dialyzed mucin, a concentration significantly higher than physiological levels, when compared to the growth of bacteria cultured in the same medium supplemented with 3% undialyzed mucin or supplemented with glucose or casamino acids [57]. Furthermore, the MB growth curve of ATCC 19606^T^ cells resembles the biphasic pattern displayed by *P*. *aeruginosa* PA14 when cultured on mucin as the sole carbon source [57]. Taken together, these observations indicate that ATCC 19606^T^ uses mucin as a nutrient source with growth responses that are similar to those described for *P*. *aeruginosa* PA14 using the same source and type of gastric mucin.

### Mucin affects the expression of genes coding for ion acquisition and utilization functions

The addition of 0.5% mucin to SB resulted in the overall repression of 42 genes predicted to code for the uptake and utilization of critical ions including the anion phosphate and the cations Cd^2+^, Co^2+^, Fe^3+^, Mg^2+^ and Zn^2+^, with iron being the most affected; the transcription levels of 25 genes coding for iron acquisition and metabolism functions were reduced significantly (Table 2).

**Table 2:** *A*. *baumannii* ATCC 19606^T^ ion metabolism/transport-associated genes with decreased expression in the presence of mucin.

Gene Identifier^#^	Fold Change	*P* value	Predicted function
***Iron acquisition and metabolism***			
A1S_0980	-2.113680918	5.00265E^-05^	Ferric enterobactin outer membrane receptor protein FepA
A1S_0981	-3.385434836	5.21485E^-06^	Ferric enterobactin outer membrane receptor protein FepA
A1S_1063	-2.016852776	0.002267793	TonB-dependent siderophore receptor protein
A1S_1359	-23.11843364	3.44387E^-19^	ABC-type Fe^3+^ transport system, periplasmic component
A1S_1360	-16.17581269	2.57193E^-16^	ABC-type Fe^3+^ transport system, periplasmic component
A1S_1361	-3.784028714	0.000130653	ABC transporter, ATP-binding protein
A1S_1362	-4.83973109	0.000161892	ABC transporter permease, 2-aminoethylphosphonate transport, Fe3+ transport
A1S_1553	-2.317106774	8.65984E^-07^	MotA/TolQ/ExbB proton channel, ExbB
A1S_1647	-2.724272435	0.000509833	Baumannoferrin biosynthesis protein, BfnA
A1S_1648	-3.276727264	0.000359948	Lysine/ornithine N-monooxygenase, baumannoferrin biosynthesis protein, BfnB
A1S_1649	-2.798094895	0.002457074	RND efflux transporter, baumannoferrin export protein, BfnC
A1S_1655	-4.491350383	2.53649E^-05^	Ferric siderophore receptor protein, baumannoferrin receptor protein, BfmH
A1S_1667	-2.101551062	6.59999E^-05^	Ferric hydroxamate siderophore receptor protein, FhuE
A1S_1785	-2.036033809	0.000581524	Iron ABC transport protein
A1S_2076	-2.290492952	0.000388929	TonB-dependent siderophore receptor protein, FhuE
A1S_2080	-2.460606915	0.000179307	TonB-dependent siderophore receptor, Cir family protein, CirA
A1S_2376	-3.48002397	0.000866488	ABC transport protein, acinetobactin secretion protein, BarA
A1S_2378	-2.056743703	0.000471467	ABC transport protein, acinetobactin secretion protein, BarB
A1S_2379	-2.471048652	0.014106234	Acinetobactin biosynthesis protein, BasG
A1S_2380	-3.043297793	0.029873076	Acinetobactin biosynthesis protein, BasF
A1S_2386	-2.638073309	0.000870159	Ferric acinetobactin periplasmic binding protein, BauB
A1S_2387	-4.349789327	0.002566617	Ferric acinetobactin transport protein, BauE
A1S_3048	-3.092680681	1.64293E^-05^	Uncharacterized small metal-binding protein
A1S_3174	-5.393271807	0.000347121	Bacterioferritin-associated ferredoxin
A1S_3339	-2.168086854	0.036495615	Ferrichrome-iron receptor
***Phosphate acquisition and metabolism***			
A1S_0462	-4.856772439	4.37285E^-05^	Phosphatase
A1S_0463	-21.07140251	3.86471E^-14^	Alkaline phosphatase
A1S_1662	-2.941837975	0.000264374	Histidine phosphatase super family protein
A1S_2445	-3.416948938	8.53187E^-07^	Phosphate import ATP-binding protein PstB
A1S_2446	-13.40837622	8.60938E^-21^	Phosphate transport system permease protein PstA
A1S_2447	-15.87820244	9.27043E^-19^	Phosphate transport system permease protein
A1S_2448	-32.11558164	2.20625E^-19^	Phosphate transport system substrate-binding protein
A1S_2677	-3.809656159	0.000110621	Alkaline phosphatase D family protein
A1S_2749	-3.021754039	0.002186855	2 phosphatidic acid phosphatase, PAP2 family protein
A1S_2821	-2.467338722	4.09571E^-05^	Alkylphosphonate uptake protein PhnA
A1S_3374	-3.202616004	5.02815E^-06^	Phosphate regulon transcriptional regulatory protein PhoB
A1S_3375	-5.249102289	9.56402E^-11^	Phosphate regulon transcriptional regulatory protein PhoB
A1S_3376	-2.212153424	1.97198E^-06^	Phosphate regulon sensor kinase PhoR
***Co/Zn/Cd transport***			
A1S_1044	-14.41769701	7.52715E^-17^	Co/Zn/Cd efflux system
A1S_1045	-6.442313497	2.08596E^-12^	Co/Zn/Cd efflux system
***Mg transport***			
A1S_2069	-7.689129605	1.35076E^-05^	MgtC Mg^2+^ family transporter
A1S_2070	-10.84105677	2.27148E^-05^	MgtA Mg^2+^ ABC transporter ATPase

^#^Genes grouped together represent gene clusters transcribed in the same direction, some of which are potential polycistronic operons, and predicted to code for related functions.

**Fig 5:**
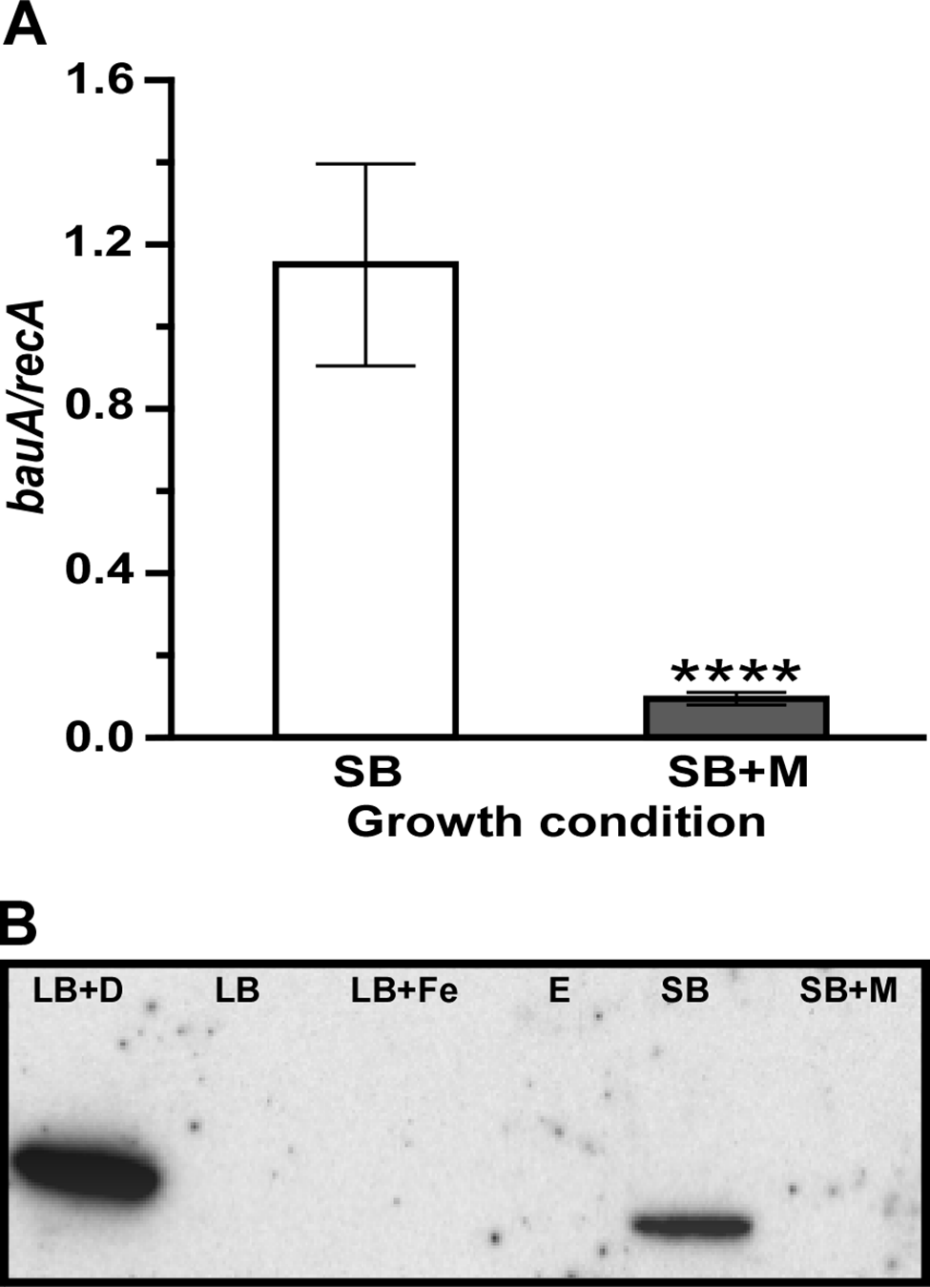
Differential expression of *bauA* in response to the presence of mucin. (A) qRT-PCR analysis of *bauA* in bacterial cells cultured in SB (white bar) or SB+M (grey bar) performed using nine replicates. Significantly different values (*P* ≤ 0.0001) are identified by four asterisks and error bars represent the standard error of each data set. (B) Western blot of whole-cell lysate proteins obtained from ATCC 19606^T^ bacterial cells grown in LB (LB), LB supplemented with 100 μM dipyridyl (LB+D) or 100 μM FeCl_3_ (LB+Fe), SB, or SB+M. E, empty lane. SDS-PAGE size-fractionated proteins were blotted onto nitrocellulose and probed with anti-BauA polyclonal antibodies. The western blot shown in this panel is a representative image of three blots obtained with three independent biological samples prepared as described above. All three blots produced the same outcome.

Mucin significantly reduced the expression of *bfnA* (A1S_1647), *bfnB* (A1S_1648), *bfnC* (A1S_1649) and *bfnH* (A1S_1655). These four genes are predicted to code for proteins involved in the biosynthesis, export and uptake of baumannoferrin, a hydroxamate siderophore produced by the *A*. *baumannii* AYE strain but not by ATCC 19606^T^ [58]. The presence of mucin also repressed the transcription of 6 of the 18 coding regions located within the *bas*-*bau* gene cluster, which is required for the active expression of the acinetobactin-mediated iron acquisition system [37, 59] and the virulence of the ATCC 19606^T^ strain [32]. Mucin significantly repressed *barA* (A1S_2376) and *barB* (A1S_2378), which code for putative acinetobactin secretion functions, as well as the transcription of the acinetobactin biosynthesis genes *basG* (A1S_2379) and *basF* (A1S_2380) (Table 2). The transcription of *bauB* (A1S_2386) and *bauE* (A1S_2387), which code for functions associated with the binding and internalization of ferric acinetobactin complexes, was also significantly reduced by mucin. Although the transcription of *bauD* (A1S_2389) and *bauA* (A1S_2385), which are part of the *bauDCEBA* locus and code for the ferric acinetobactin permease and outer membrane receptor protein, respectively, were reduced more than 2-fold, the transcriptional differences between the two experimental conditions were not statistically significant according to the conditions used to compare transcripts differentially produced in response to mucin and were therefore not included in Table 2. However, further analysis by qRT-PCR confirmed that transcription of *bauA* was down-regulated significantly (*P* < 0.0001) in the presence of mucin (Fig 5A). As previously demonstrated [37], the 84-kDa BauA protein was detected by western blot in ATCC 19606^T^ cells cultured under iron-chelated conditions, but was absent when cells were grown in either LB broth or LB broth supplemented with 100 μM FeCl_3_ (Fig 5B). Furthermore, BauA was present in cell lysates obtained from bacteria cultured in SB but absent in lysates of bacteria cultured in SB+M (Fig 5B). Thus, the transcription of *bauA* and the consequent production of the acinetobactin outer membrane receptor protein BauA are indeed significantly inhibited by the presence of mucin.

Mucin also reduced the transcription of genes encoding uncharacterized iron permeases (A1S_1359 and A1S_1362) as well as putative receptors for xenosiderophores including ferric enterobactin (A1S_0980/A1S_0981), ferric hydroxamates (A1S_1667, A1S_2076), ferrichrome (A1S_3339) and a CirA ortholog (A1S_2080) (Table 2). The transcription of A1S_1785, which could code for an iron ABC transporter, is also significantly reduced by mucin. Also down-regulated, although less than 2-fold, was the expression of A1S_1787, which codes for the periplasmic component of an ABC ferric hydroxamate transporter (Table 2). Interestingly, A1S_1785 and A1S_1787 flank the A1S_1786 gene, which is predicted to code for a FepD ortholog involved in periplasmic iron transport functions, with all of them transcribed in the same direction and having the potential of being an operon involved in iron acquisition. All these results suggest that mucin contains readily available iron that bacteria can use without expressing high-affinity transport systems. This possibility was confirmed by the observation that the addition of 0.5% mucin to SB significantly increased (*P* ≤ 0.05) the iron concentration from 20.1± 0.6233 μg/l to 219.3 ± 2.644 μg/l. This 10.9-fold increase in iron concentration explains the lack of detection of BauA in cells cultured in SB+M (Fig 5B).

In addition to iron-related functions, mucin also negatively affected the expression of genes coding for proteins potentially associated with the binding and transport of other metals including Co^2+^, Zn^2+^ and Cd^2+^ (A1S_1044 and A1S_1045) as well as Mg^2+^ (A1S_2069 and A1S_2070) (Table 2). This negative effect, which ranged between 6- and 14-fold negative changes in the transcription of these genes, could be explained by the significantly (*P* ≤ 0.05) increased concentration of Co^2+^ (from 2.72 ± 0.1666 μg/l to 3.89 ± 0.1541 μg/l), Zn^2+^ (from 123.104 ± 1.559 μg/l to 183.77 ± 2.388 μg/l) and Mg^2+^ (from 305.934 ± 3.257 μg/l to 643.561 ± 3.602 μg/l) upon the supplementation of SB with 0.5% mucin. In contrast, the addition of mucin did not cause significant changes in Cd^2+^ concentration, which was detected at a level of 0.661 ± 0.07093 μg/l in SB.

The presence of mucin also affects the expression of 13 predicted genes involved in bacterial phosphate metabolism (Table 2). It down-regulates the expression of genes coding for phosphatase activity (A1S_0462, A1S_0463, A1S_2677, A1S_2749), as well as the predicted operons A1S_2445-A1S_2448 and A1S_3374-A1S_3376, which code for potential phosphate transport and regulatory functions, respectively.

In summary, the RNA-Seq data are consistent with mucin being a source of different chemical elements that are critical nutritional requirements for bacterial growth.

### Mucin induces the expression of genes associated with bacterial virulence

**Fig 6:**
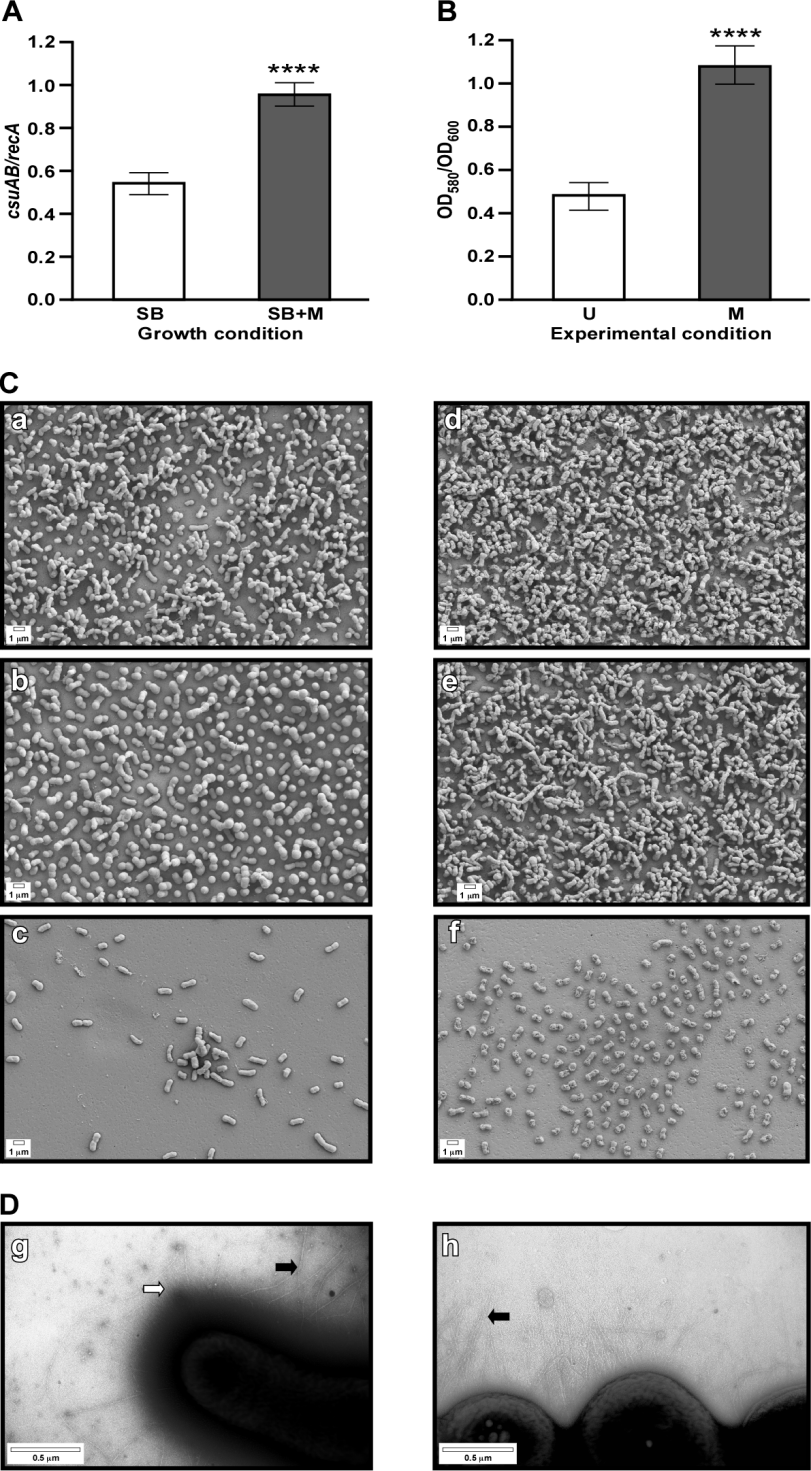
Effect of mucin on the expression of the *csuAB* gene, biofilm formation on plastic and pili production. (A) qRT-PCR analysis of *csuAB* in ATCC 19606^T^ cells cultured in SB (white bars) or SB+M (grey bars). (B) Crystal violet staining of biofilms formed by ATCC 19606^T^ on uncoated (U, white bar) or mucin-coated (M, grey bar) polystyrene tubes. qRT-PCR and biofilm analyses were performed using nine and six replicates for each study, respectively. Significantly different values (*P* ≤ 0.0001) are identified by four asterisks and error bars represent the standard error of each data set. (C) SEM of biofilms formed on uncoated (a, b, c) or mucin coated (d, e, f) coverslips by ATCC 19606^T^ cells. Micrographs were taken above (a and d), at (b and e), or below (c and f) the liquid-air interface at a magnification of 10,000x. Scale bars, 1 μm. (D) TEM of bacterial cells lifted from the surface of SA (g) or SA supplemented with mucin (h). Micrographs were taken at a magnification of 30,000x. Scale bars, 0.5 μm.

The RNA-Seq data also showed the differential transcription of genes coding for proteins known to be involved in virulence and the interaction of bacteria with the environment. Specifically, the transcription of four (A1S_2213-A1S_2215 and A1S_2218) of the six (A1S_2213-A1S_2218) genes that code for the CsuAB/A/B/C/D/E chaperone-usher system, which is responsible for the assembly of type I pili produced by the ATCC 19606^T^ strain [34], was significantly up-regulated in the presence of mucin (Table 3). qRT-PCR analysis of *csuAB* (A1S_2218) showed that the presence of 0.5% mucin in SB indeed significantly increased (*P* < 0.0001) the expression of this gene coding for the main pilin subunit (Fig 6A).

**Table 3:** *A*. *baumannii* ATCC 19606^T^ virulence-associated genes with increased expression in the presence of mucin.

Gene Identifier^#^	Fold Change	*P* value	Predicted function
A1S_0690	2.684637704	1.05547E^-05^	Pilus subunit protein FilA
A1S_1193	2.480689253	0.000532076	OmpA/MotB protein
A1S_1288*	2.081708589	1.3823E^-05^	Type VI secretion system protein, VGR-like protein, TssI
A1S_1289*	2.020380724	4.1958E^-05^	Type VI secretion system protein, VGR-like protein, TssI
A1S_1292	2.014662816	0.003396271	Uncharacterized protein
A1S_1293*	2.587613744	0.000203864	Type VI secretion protein, TssB
A1S_1294*	3.409115093	3.63899E^-06^	Type VI secretion protein, TssB
A1S_1295	4.153898235	1.80402E^-07^	Type VI secretion protein, TssC
A1S_1296	5.765082934	1.10125E^-08^	Type VI secretion system effector protein Hcp1, TssD
A1S_1297	2.844802691	1.09366E^-11^	Type VI secretion system lysozyme-like protein, TssE
A1S_1298*	2.106834338	0.000245984	Type VI secretion system protein ImpG, TssF
A1S_1299*	3.805552399	1.07029E^-10^	Type VI secretion system protein ImpG, TssF
A1S_1301	2.843789765	7.87924E^-09^	Uncharacterized membrane protein
A1S_1302*	2.434126602	7.89556E^-08^	Type VI secretion protein, TssM
A1S_1303*	2.246292519	2.32718E^-05^	Type VI secretion protein, TssM
A1S_1304	2.055714347	0.000201054	Type VI secretion protein, TagF
A1S_1305	2.178282322	8.78075E^-06^	Type VI secretion protein, TagN
A1S_1306	2.483203275	4.37454E^-05^	PAAR domain containing protein
**A1S_1307**	**2.14514296**	**1.82021E**^**-06**^	**Type VI secretion ATPase, TssH**
A1S_1309	2.249010853	3.32742E^-07^	Type VI secretion protein, TssK
A1S_2213	2.715635166	1.95526E^-10^	Pili protein, CsuE
A1S_2214	3.138205041	1.23081E^-14^	Pili usher protein CsuD
A1S_2215	3.047452851	1.09467E^-08^	Pili assembly chaperone protein, CsuC
A1S_2218	2.152070566	0.012039198	Pili protein, CsuA/B

^#^Genes grouped together represent gene clusters transcribed in the same direction, some of which are potential polycistronic operons, and predicted to code for related functions.

*Gene identifiers that could represent a potential single coding region due to DNA sequencing errors.

Bolded gene identifier, differential expression tested by qRT-PCR.

Because the *csu* operon is essential for biofilm formation by *A*. *baumannii* ATCC 19606^T^ on plastic surfaces [34], we tested whether the increased transcription of *csuAB* corresponded to increased biofilm formation in response to the presence of mucin. Crystal violet assays using either uncoated or mucin-coated polystyrene tubes demonstrated that ATCC 19606^T^ bacteria formed a significantly higher (*P* = 0.0001) amount of biofilm in the presence of mucin when compared with the amount of biofilm formed in the absence of mucin (Fig 6B). SEM analysis revealed that ATCC 19606^T^ bacteria formed more biofilm below and, particularly, at and above the liquid-air interface on the surface of mucin-coated plastic coverslips when compared with uncoated coverslips (Fig 6C). Considering the role pili play in biofilm biogenesis by ATCC 19606^T^ [34], we determined whether the presence of mucin altered the presence of pili on the surface of bacterial cells. TEM analysis of bacteria lifted from the surface of SA plates showed the presence of both short pili (white arrow) as well as long pili (black arrow) on the cell surfaces (Fig 6Dg). However, only the long pili were detected on the cell surface of bacteria lifted from SA supplemented with 0.5% mucin (Fig 6Dh). Taken together, these findings suggest that the presence of mucin not only increases biofilm biogenesis but also promotes the production of more than one type of pilus, an observation that could be related to the increased transcription of A1S_0690. This gene codes for a FilA homolog that could be part of a type III pilus [60], the role of which remains to be examined experimentally. The significant transcriptional induction of A1S_1193, coding for OmpA, which plays a key role in the ability of ATCC 19606^T^ to interact with abiotic and biotic surfaces [23], further supports the effect of mucin on the interaction of bacteria with different substrates as shown in Figs 1A and 6C.

**Fig 7:**
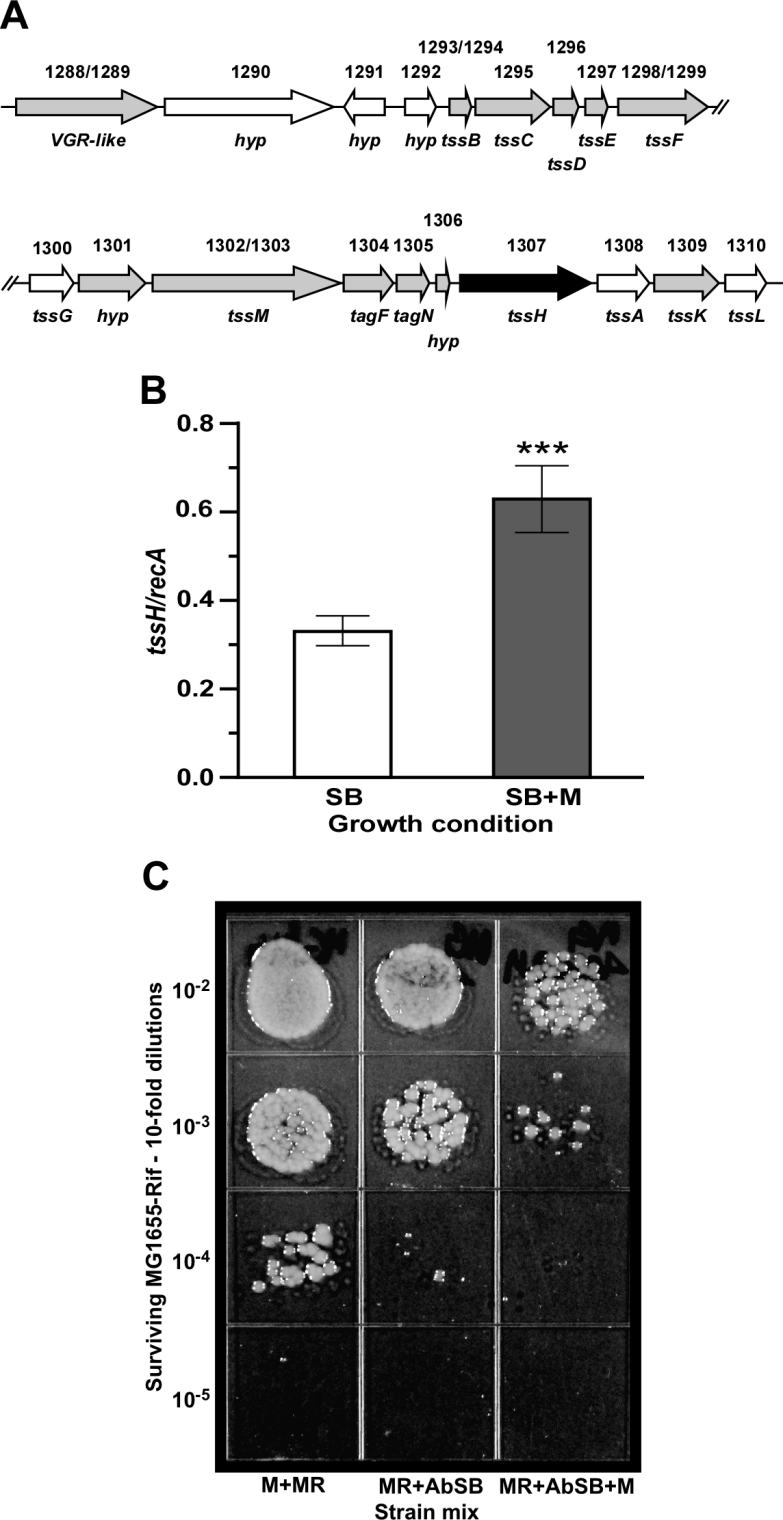
Differential transcription of genes coding for T6SS components in response to the presence of mucin. (A) ATCC 19606^T^ genes coding for T6SS components. The arrows represent each coding region and its direction of transcription. The white and grey arrows represent genes constitutively and differentially expressed in response to the presence of mucin, respectively. The black arrow indicates *tssH* (A1S_1307), which was used to confirm the mucin-mediated up-regulation effect by qRT-PCR. Numbers above the arrows represent cognate A1S_ gene annotation numbers, and gene names are indicated below each arrow. Coding regions A1S_1288/1289, A1S_1293/1294, A1S_1298/1299, and A1S_1302/1303 are each represented as a single coding region since potential nucleotide sequencing errors could have resulted in the annotation of multiple open reading frames. *hyp*, gene coding for a hypothetical protein. (B) qRT-PCR analysis of the differential expression of A1S_1309 in bacterial cells cultured in SB (white bar) or SB+M (grey bar) was performed using nine replicates. Significantly different values (*P* ≤ 0.001) are identified by three asterisks and error bars represent the standard error of each data set. (C) Bacterial killing assay using *E*. *coli* MG1655-Rif (MR) as a prey and ATCC 19606^T^ cultured in SB (AbSB) or SB+M (AbSB+M) as a predator. Representative image of experiments performed two times using three different biological samples for each experiment (*n* = 6) showing differences in the amount of bacterial colonies formed on the surface of the agar plate among the tested samples.

Along with increased transcription of genes coding for proteins needed for adherence and biofilm formation, mucin induced the transcription of genes encoding the components of the type VI secretion system (T6SS) described in the *A*. *baumannii* ATCC 19606^T^, ATCC 17978 and *Acinetobacter nosocomialis* M2 clinical isolates as well as other *Acinetobacter* species [61, 62]. RNA-Seq showed that the addition of mucin resulted in a significant up-regulation of 13 genes (Table 3) belonging to an approximately 22-kb gene cluster that codes for VgrG (TssI) and other T6SS orthologs as well as the uncharacterized proteins A1S_1292, A1S_1301 and A1S_1306, which could play a role in the function of this secretion system (Table 3 and Fig 7A). Although most of the *tss* genes were induced between 2- and 4-fold, *tssC* and particularly *tssD*, which codes for the secreted hemolysin-co-regulated protein (Hcp), were the most mucin up-regulated genes with 4.1- and 5.7-fold changes, respectively. These variations in differential expression are in accordance with the proposal that not all T6SS components are equally produced since some of them are intracellular structural proteins that could be recycled, while others are consumed secreted proteins, such as TssD, that must be produced at higher levels [63]. The qRT-PCR analysis of A1S_1307, which codes for the TssH ATPase protein, showed that the presence of mucin significantly increased (*P* < 0.0006) the transcription of this gene when compared with transcription levels in cells cultured in the absence of mucin (Fig 7B). This analysis not only confirmed the transcriptomic data, but also showed that *tssH* is transcribed in the absence of mucin, albeit at significantly lower levels. Interestingly, the transcription of A1S_1300, A1S_1308 and A1S_1310, which code for the TssG, TssA and TssL orthologs, respectively (Fig 7A) was not significantly affected by the presence or absence of mucin in the culture medium.

The biological effect of the mucin-induced expression of the T6SS genes was tested with bacterial killing assays using an approach similar to that recently described [38]. Fig 7C shows that ATCC 19606^T^ bacteria cultured in SB resulted in readily detectable killing activity when co-cultured with the *E*. *coli* MG1655-Rif derivative as compared to the *E*. *coli* MG1655/MG1655-Rif mixture, which was used as a negative control. This figure also shows that the co-incubation of *E*. *coli* MG1655-Rif with ATCC 19606^T^ cells cultured in SB+M resulted in a significant and apparent increase in killing activity at all tested dilutions when compared with the killing activity displayed by ATCC 19606^T^ cells cultured in SB.

Taken together, the finding that pili and biofilm biogenesis as well as the expression of genes coding for T6SS functions and the biological activity of this secretion system are significantly enhanced in the presence of mucin directly supports the potential role of this host product in the virulence of *A*. *baumannii*.

## Discussion

### Mucin and *A*. *baumannii* virulence

Previous studies have described the role that the overproduction of mucin plays in the regulation of the host inflammation response, which leads to a decreased resistance to bacterial pathogens, such as *E*. *coli*, *Staphylococcus aureus*, *Helicobacter pylori* and *P*. *aeruginosa*, and an increase in the virulence of these pathogens [17, 18]. However, almost nothing is known about the role mucin plays in *A*. *baumannii*. In this study we uncovered the function of mucin as an extracellular signal that triggers the expression of virulence-associated genes aiding in *A*. *baumannii’*s persistence and pathogenicity. RNA-Seq data show that the presence of mucin increased transcription of eight genes coding for proteins involved in phenylacetate degradation (Table 1 and Fig 3). Furthermore, our qRT-PCR transcriptional analysis showed that *paaH*, one of the eight differentially transcribed genes, was poorly expressed in the absence of mucin (Fig 3B), an observation that indicates that this glycoprotein indeed acts as an inducer for these genes. However, our data also show that although the presence of mucin significantly increased the expression of the *paaF*-*paaI* genes, which are the last eight coding regions of the *paaF*-*paaI* gene cluster, it did not significantly affect the expression of the *paaA-paaE* genes, which are the first five components of this operon (Fig 3A). These observations indicate that not all components in this gene cluster, which is predicted to be a polycistronic operon [64], are equally responsive to mucin. Thus, the differential expression of this operon in response to external signals is likely complex. It is possible that other internal promoter elements, in addition to that predicted to be upstream of *paaA* and driving the expression of all 13 coding regions under the control of the GacS/GacA two component regulatory system [64], could control the overall expression of this operon. It is also possible that the first part of the transcript produced by this operon is susceptible to degradation when bacterial cells are cultured in the presence of mucin, possibilities that remain to be tested experimentally.

The biological role and relevance of the *paa* operon and its products is reflected not only by their capacity to provide nutrients to bacterial cells during infection as shown by our data, but also by producing intermediate metabolites, such as a ring-1-2-epoxide and its phenolic breakdown product 2-hydroxyphenylacetate, which contribute to bacterial virulence because of their host toxicity [64]. Products of the Paa pathway may also be involved in the reduction of reactive oxygen species in neutrophils [65], which may link the overexpression of this pathway with increased virulence when mucin is present in the extracellular environment. A recent study describing a zebrafish infection model has demonstrated that the deletion of *paaA* leads to the accumulation of phenylacetate, which ultimately serves as a chemoattractant that causes the recruitment of neutrophils to the infection site and a consequent reduction in bacterial burden and virulence [66]. Interestingly, inactivation of *gacS*, which codes for a sensor kinase that modulates the expression of the *paa* operon [64], produced a defective virulence response similar to that detected in the zebrafish model using an *A*. *baumannii* ATCC 17978 *paaA* derivative. This result indicates that the GacA/GacS two-component regulatory system plays a key role in coordinating the expression of bacterial functions, including the expression of the *paa* operon, which are critical in host-pathogen interactions that result in host infection. However, the signal sensed and transduced by this transcriptional regulatory system has not been identified. Based on these observations and our data showing increased expression of the *paa* operon in the presence of mucin, it is possible to speculate that this host-produced glycoprotein could be one of the environmental signals that controls the expression of a novel *A*. *baumannii* virulence function that results in a decrease host immune response.

In addition to the *paa* gene cluster, mucin up-regulated 13 genes related to the production of a T6SS (Fig 7A). This contact-dependent secretion system plays a critical role in bacterial antagonistic responses and host-pathogen interactions, all of which are of great importance for diverse bacteria to successfully persist in different ecosystems and cause host infections [63, 67]. All sequenced *A*. *baumannii* strains as well as non-pathogenic *Acinetobacter* strains show the presence of core T6SS genes [62]; however, not all *A*. *baumannii* isolates are able to secrete the Hcp effector protein associated with this secretion system, and therefore produce functional T6SS [62]. A recent study of the ATCC 17978 and Ab_04_ clinical isolates showed that although both strains have all genes needed to assemble an active T6SS, their capacity to kill unrelated bacteria depends on the presence of the pAB3 and pAB04-1 conjugative resistance plasmids, respectively [38]. These plasmids code for the TetR1 and TetR2 repressors that control the expression of T6SS in these strains. These findings [38] not only elucidated the variability of the T6SS-mediated killing phenotype among different *A*. *baumannii* isolates, but also lead to the conclusion that strains such as ATCC 19606^T^ express a constitutively active secretion system since it lacks the *tetR1* and *tetR2* genes [38]. In addition to matching these observations, our work adds the new finding that the presence of mucin in the medium significantly enhances the expression of 12 of the 15 genes located within the *tssB-tssL* operon as well as the transcription of the *vgrG* ortholog located nearby this chromosomal region (Fig 7A). Interestingly, the transcription of *tssG*, *tssA* and *tssL* was not significantly affected by the presence of mucin in the extracellular environment. This finding together with the different transcriptional responses of the *tss* genes to the presence of mucin suggest that more than one promoter element could control the expression of this operon, a possibility that remains to be experimentally explored. Our results also provide evidence supporting the role of mucin as a regulatory factor controlling T6SS expression, in addition to intracellular and extracellular factors already described, including changes in extracellular phosphate and iron concentrations [63, 68]. The expression of the *P*. *aeruginosa* H2-T6SS, which modulates the interaction of this pathogen with eukaryotic host cells, is repressed by high iron concentrations *via* a Fur-mediated process [69]. High levels of iron and inorganic phosphate (P_i_) repress the expression of the *Edwardsiella tarda* T6SS through a regulatory circuit that involves the interaction of the PhoB-PhoR and Fur transcriptional regulators [70]. It is of note that these two latter observations appear not to match our data showing that although mucin contains enough iron to repress the expression of the *bauA* iron-regulated gene, the presence of this host glycoprotein in the medium significantly increased the expression of *tss* genes. A similar conclusion could be drawn regarding the role of phosphate since the presence of mucin significantly reduced the transcription of genes coding for phosphate transport as well as the *phoB* and *phoR* genes (Table 2), which code for the two-component regulatory system that senses changes in extracellular inorganic phosphate [71], but increased the transcription of ATCC 19606^T^ *tss* genes. Thus, our observations reveal the complexity of the factors and signals that control the expression of this critical bacterial secretory function by mechanisms that are poorly understood. Based on these observations, it is tempting to speculate that mucin favors the interaction of *A*. *baumannii* with host mucin-coated surfaces by increasing T6SS activity, a response that ultimately results in bacterial persistence and the pathogenesis of host infections. Such a possibility is strongly supported by the observation that mucin activates the T6SS expressed by *Vibrio cholerae* pandemic strains, when tested *in vitro* using bovine submaxillary mucins or *in vivo* in an infant mouse model [72]. Interestingly, the regulatory effect of mucin on *V*. *cholerae* is further modulated by the action of bile salt metabolites, a response that is critical for the interaction of this pathogen with the intestinal commensal microbiota as well as the pathogenesis of diarrheal disease. Taken together, all these observations indicate that host environmental signals play a critical role in the expression and activity of bacterial T6SS.

The effect of mucin on the production of bacterial surface proteins and appendages that play critical roles in host-pathogen interactions is further underscored by the differential expression of genes coding for an uncharacterized type III pilus and OmpA (Table 3). These factors are involved in *A*. *baumannii* biofilm formation and interaction with eukaryotic cells [23, 60, 73]. It is of note that the presence of mucin also stimulates the production of different types of pili, some of which have yet to be characterized (Fig 6Dg and 6Dh). This finding is in agreement with our previous observation that an ATCC 19606^T^ *csuE* isogenic mutant, which produces neither CsuAB/ABCDE pili nor biofilms on plastic, adheres to epithelial cells and produces uncharacterized pili when cultured in the presence of sheep erythrocytes [34, 74]. Considering all these observations, we propose that the concerted action of several bacterial factors such as the OmpA adhesin, different pili, including type I, and a T6SS, which could control type I pili production [60], determine the virulence and interaction of *A*. *baumannii* with the host when this pathogen encounters a mucin-rich environment.

It is important to note that all interactions described above could be affected not only by mucin-regulated bacterial products, but also mucin properties such as glycosylation. The BabA-dependent binding of *Helicobacter pylori* to MUC5AC isolated from Fut2 knock-out mice is impaired due to a drastic reduction in fucosylation when compared to MUC5AC produced by normal animals [75]. This is a possibility that could be tested once the molecular nature of the *A*. *baumannii*-mucin interactions is elucidated. The source and composition of mucins could be an additional factor that may influence the outcome of mucin-pathogen interactions. Our finding and that reported by Bachmann *et al*. [72] showing that PGM and bovine submaxillary mucin, both from Sigma-Aldrich, increase the T6SS activity expressed by *A*. *baumannii* and *V*. *cholerae*, respectively, suggest that this particular bacterial response is not affected by potential differences between these two types of mucins. In contrast, the relationship between the nature and source of mucins and the capacity of pathogens such as *P*. *aeruginosa*, a respiratory pathogen that has been widely used for this purpose, to form biofilms on different substrates is less apparent. As we observed with *A*. *baumannii* using commercial PGM, the coating of abiotic surfaces with mucin purified from Calu-3 cells allowed *P*. *aeruginosa* PAO1 to form more abundant and denser biofilms when compared to untreated substrates [76]. On the other hand, the addition of 0.5% untreated commercial PGM to artificial sputum medium [47] or LB broth [45] or 0.5% purified PGM predominantly containing MUC5AC [44] prevented *P*. *aeruginosa* PAO1 to adhere to solid surfaces and develop biofilm structures. Altogether, these observations indicate that bacterial responses could be affected by the nature of the mucin present in the extracellular media, an outcome that is further compounded by the different culture and experimental conditions used in the studies cited above. In addition, the advantage of using purified mucin components in some of the studies cited above is questionable not only because purified mucins display properties different from the parent mucus sample, but also the fact that mucins could be the product of more than 20 genes belonging to the human *MUC* gene family [77]. Taking into account these observations, it is possible to speculate that the biological effect of mucins is most likely due to the combination of several products rather than a single one, which most likely does not reflect the biological properties of the natural source. Unfortunately, we could not find systematic studies that examined the role of different mucins in bacterial responses under the same experimental conditions. The data and observation described in this report should facilitate the development of these studies with *A*. *baumannii*.

### Mucin as a nutrient source

Our RNA-Seq data also indicate that a mucin-rich environment serves as a source of nutrients that *A*. *baumannii* needs to persist and prosper in the host during infection. The finding that 24 genes coding for iron acquisition and metabolic functions are significantly down-regulated when mucin is present (Table 2) strongly supports this possibility. The inhibitory effect is exerted not only on genes that code for the production and uptake of acinetobactin (Table 2 and Fig 5), the only fully active siderophore-mediated system expressed by the strain ATCC 19606^T^ [37], but also for the utilization of xenosiderophores. The latter category includes the catechol siderophore enterobactin and the hydroxamate baumannoferrin, which could both be utilized by ATCC 19606^T^ even though this strain does not produce them. Altogether, these findings suggest that mucin provides an iron-rich environment and therefore the expression of iron acquisition genes, mainly those coding for high-affinity siderophore-mediated systems, are not necessary when *A*. *baumannii* encounters a mucin-rich environment. Such a possibility is supported not only by our data showing that the addition of mucin increases more than 10-fold the iron content of SB, but also by a previous study showing the ability of mucin to bind radioiron with relatively low affinity (Kd of 9.09x10^-5^) but high capacity, a property displayed by mucins obtained from different sources including rat duodenum, calf intestine and bovine stomach and submaxillary glands [78]. Accordingly, it was observed that *Neisseria meningitidis*, a pathogen that does not express siderophore-mediated iron acquisition systems, is able to acquire iron from hog gastric mucin, even after it was extensively dialyzed against deionized water to remove free-iron contaminant, when cultured in iron-limited media [79]. Taken together, these results demonstrate that mucin is an iron source that *A*. *baumannii* can use without the involvement of high-affinity iron chelators as well as through non-heme mediated pathways during the initial host-pathogen interactions. However, the following progression and dissemination steps of the infection process occur only if this pathogen expresses an active high-affinity iron acquisition system as demonstrated using different virulence models [32]. Furthermore, the report that mucin is also a Co^2+^ and Zn^2+^ source [78] and our findings regarding the Co^2+^ and Zn^2+^ content of mucin also explains the significant reduction in the expression of A1S_1044 and A1S_1045 (Table 2), both of which code for predicted efflux systems involved in the transport of these metals. The concerted reduction in the expression of genes coding for phosphate utilization and transport, as well as regulators that control these functions (Table 2), could reflect the presence of phosphate in modified sialic acids, monosaccharides normally found in mucin [80]. Thus, the production and secretion of *A*. *baumannii* glycosidases, which remain to be identified, could result in the degradation of phosphorylated sialic acids and the consequent increase in P_i_ available to bacteria encountering mucin in the extracellular environment.

The significantly enhanced transcription of genes coding for the metabolism and transport of amino acids and peptides in response to the presence of mucin (Table 1) suggests that *A*. *baumannii* uses these molecules as carbon and nitrogen sources for the biosynthesis of critical molecular components. Similarly, the increased expression of genes coding for the metabolism of catechols and benzoate (Table 1 and Fig 2) indicate that *A*. *baumannii* assimilates and metabolizes these compounds via the catechol branch of the β-ketoadipate pathway that are finally used for energy production through the TCA cycle [48, 81]. The increased expression of 27 genes related to lipid transport and metabolism (Table 1) could be explained not only by the finding that mucin contains various amounts of covalently linked lipids [78], but also the potential capacity of *A*. *baumannii* to produce and catabolize SCFAs when cultured in the presence of mucin as evidenced by the differential production of PrpB and AcsA orthologs. These enzymes, which are involved in bacterial SCFA catabolism [52–55], play a role in the catabolism of propionate and acetate, respectively, when *P*. *aeruginosa* prospers in the presence of mucin-fermenting bacteria that promote the production of these SCFAs [56]. Mucus glycoprotein isolated from rat salivary glands contains 0.3% and 18.4% covalently bound and associated fatty acids, respectively [82]. Furthermore, the latter components include 17.9% phospholipids, whose degradation could also contribute to the phosphate pool available to bacteria located in a rich-mucin environment.

The RNA-Seq data suggesting that mucin serves as a source of nutrients is supported by the ability of ATCC 19606^T^ to grow in broth containing only 0.5% mucin, although to lower levels when compared to SB without or with 0.5% mucin (Fig 4D). This growth response with a biphasic growth pattern mimics the ability of *P*. *aeruginosa* to grow in a chemically defined medium supplemented with 3% dialyzed and clarified PGM obtained from the same commercial source as ours [56]. The observation that *A*. *baumannii* degrades mucin as detected when cultured on the surface of a SA plate containing 0.5% mucin or by the significant reduction of protein- or carbohydrate-labeled mucin derivatives caused by bacterial cells or culture supernatants (Fig 4) further supports the capacity of *A*. *baumannii* to use mucin as a nutrient source. To the best of our knowledge, there are no reports describing the degradation of mucin and its use as a nutrient source by *A*. *baumannii*, and therefore the mechanisms by which this is achieved are mostly unknown. Mucin degradation by *P*. *aeruginosa* depends on the production of proteases, which aid in destruction of the mucin barrier and the acquisition of nutrients, an activity that is inhibited by the presence of several serine protease inhibitors [19, 83]. It is of note that our RNA-Seq data show that two ATCC 19606^T^ genes coding for proteases were up-regulated in the presence of mucin: A1S_0477, which codes for a serine protease named ClpX; and A1S_2296, which codes for a collagenase-like protease (Table 1). Interestingly, the gene that codes for the proteolytic subunit of ClpXP, ClpP (A1S_0476), is necessary for persistence of *A*. *baumannii* within the lung [84]. A previous study has also demonstrated that *Clostridium difficile* utilizes extracellular hydrolytic enzymes, such as collagenases, to obtain nutrients from mucin [85]. Therefore, the overexpression of A1S_0477 and A1S_2296 may represent some of the *A*. *baumannii* proteins that could participate in mucin degradation, a process that likely provides nutrients and favors *A*. *baumannii* growth when this pathogen interacts with mucin-covered host epithelial surfaces.

In summary, the adaptation of PGM-based protocols used to study the respiratory pathogen *P*. *aeruginosa* produced data indicating that growth of *A*. *baumannii* in the presence of mucin prior to infection is sufficient to positively affect *A*. *baumannii*’s virulence. Thus, the capacity of mucin to decrease host resistance combined with the ability of *A*. *baumannii* to sense the presence of mucin and up-regulate virulence factors, as well as to utilize mucin as a source of energy seem to play an important role in the development of severe infections associated with this pathogen. This worldwide human health problem is further compounded by the ability of mucin to bind antibiotics, such as colistin, therefore reducing the efficacy of antibiotics used to treat *A*. *baumannii* infections [86]. All these findings provide strong support for the hypothesis that the success of *A*. *baumannii* as a pathogen relates to its capacity to code for diverse metabolic and survival functions that allow it to thrive and persist in the hostile environment imposed by the human host [87]. Developing a further understanding of how *A*. *baumannii* interacts with the host environment, such as the interactions observed in this work between mucin and *A*. *baumannii* ATCC 19606^T^, may lead to the development of much needed novel treatments for infections with this pathogen, particularly those caused by MDR isolates.

## Supporting information

S1 Fig Analysis of gene expression in response to the presence of mucin in the culture medium. Overall gene expression in each of the three libraries constructed using total RNA isolated from bacteria cultured in SB (red) or SB+M (blue) was compared. After expression values are estimated, the mean expression in RNA samples obtained from bacteria cultured in SB+M mucin is slightly greater and with more variation than RNA samples obtained from bacteria cultured in SB, as seen above. All samples with similar treatment show practically identical variance and the middle 50% of all data sets overlap. Considering these facts and that the EdgeR analysis normalizes the original data, the samples are suitable for the differential gene expression analysis.
(TIF)

S2 Fig Analysis of genes differentially expressed in response to the presence of mucin in the culture medium. MDS plot of genes differentially expressed in each of the libraries prepared using total RNA isolated from bacteria cultured in SB or SB+M. Blast2GO was used to perform MDS analysis resulting in clustering based on growth in the presence of mucin (blue) or without mucin (red). This shows a difference at the transcriptional level in response to the presence of mucin and supports further analysis.
(TIF)

S3 Fig Analysis of genes differentially expressed in response to the presence of mucin in the culture medium. Volcano plot showing the overall differential gene transcription in bacteria cultured in SB vs. SB+M. Blast2GO was used to generate the volcano plot based on EdgeR values.
(TIF)

S4 Fig Analysis of genes differentially expressed in response to the presence of mucin in the culture medium. (A) Functional distribution of the 427 predicted protein-coding genes differentially transcribed in cells cultured in SB and SB+M. (B) Gene ontology (GO) analysis of the mucin-regulated genes using Blast2GO.
(TIF)

S1 Table Primers used in this work. (DOCX)

S2 Table Quality data collected from sequencing cDNA libraries constructed using RNA isolated from ATCC 19606^T^ cells cultured in SB or SB+M. (DOCX)

S3 Table *A. baumannii* ATCC 19606^T^ gene up-regulated by the presence of 0.5% mucin in swimming broth. (DOCX)

S4 Table *A. baumannii* ATCC 19606^T^ gene down-regulated by the presence of 0.5% mucin in swimming broth. (DOCX)
