# Supplementary figures and images for: Mucin acts as a nutrient source and a signal for the differential expression of genes coding for cellular processes and virulence factors in Acinetobacter baumannii
Source: PLoS One. 2018 Jan 8;13(1):e0190599. doi: 10.1371/journal.pone.0190599 (PMC5757984; doi:10.1371/journal.pone.0190599)

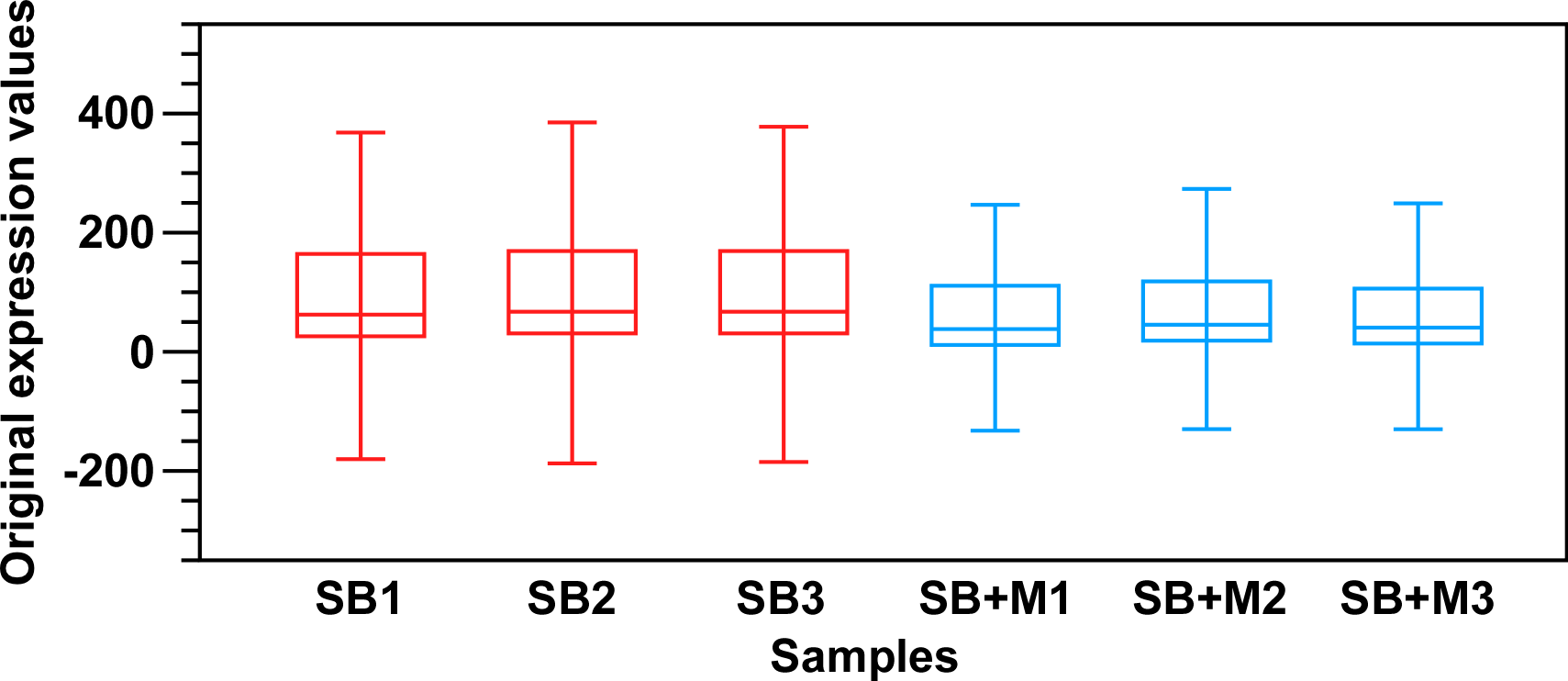

Supplement: S1 Fig — Overall gene expression in each of the three libraries constructed using total RNA isolated from bacteria cultured in SB (red) or SB+M (blue) was compared. After expression values are estimated, the mean expression in RNA samples obtained from bacteria cultured in SB+M mucin is slightly greater and with more variation than RNA samples obtained from bacteria cultured in SB, as seen above. All samples with similar treatment show practically identical variance and the middle 50% of all data sets overlap. Considering these facts and that the EdgeR analysis normalizes the original data, the samples are suitable for the differential gene expression analysis. (TIF) [file pone.0190599.s001.tif]

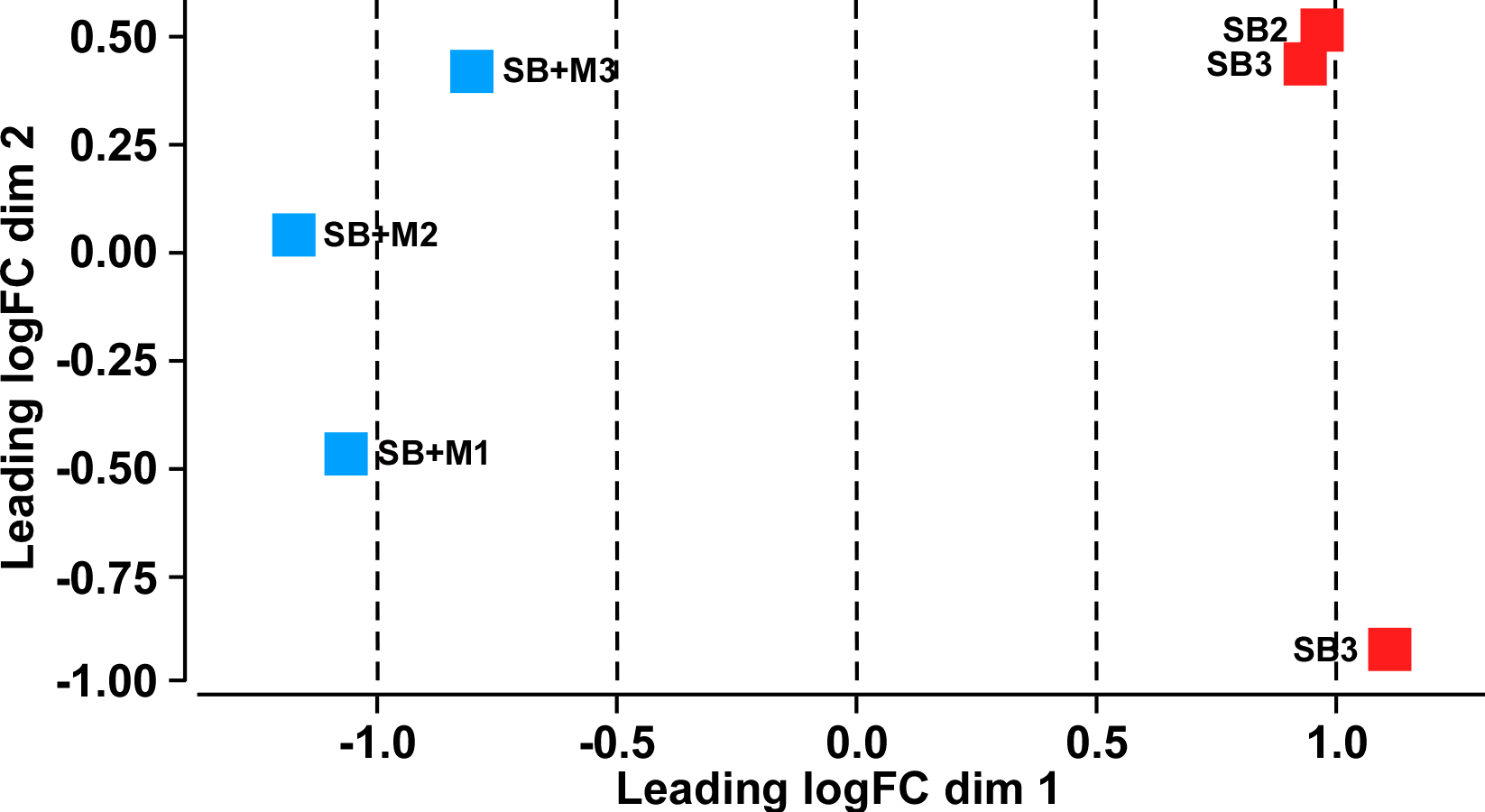

Supplement: S2 Fig — MDS plot of genes differentially expressed in each of the libraries prepared using total RNA isolated from bacteria cultured in SB or SB+M. Blast2GO was used to perform MDS analysis resulting in clustering based on growth in the presence of mucin (blue) or without mucin (red). This shows a difference at the transcriptional level in response to the presence of mucin and supports further analysis. (TIF) [file pone.0190599.s002.tif]

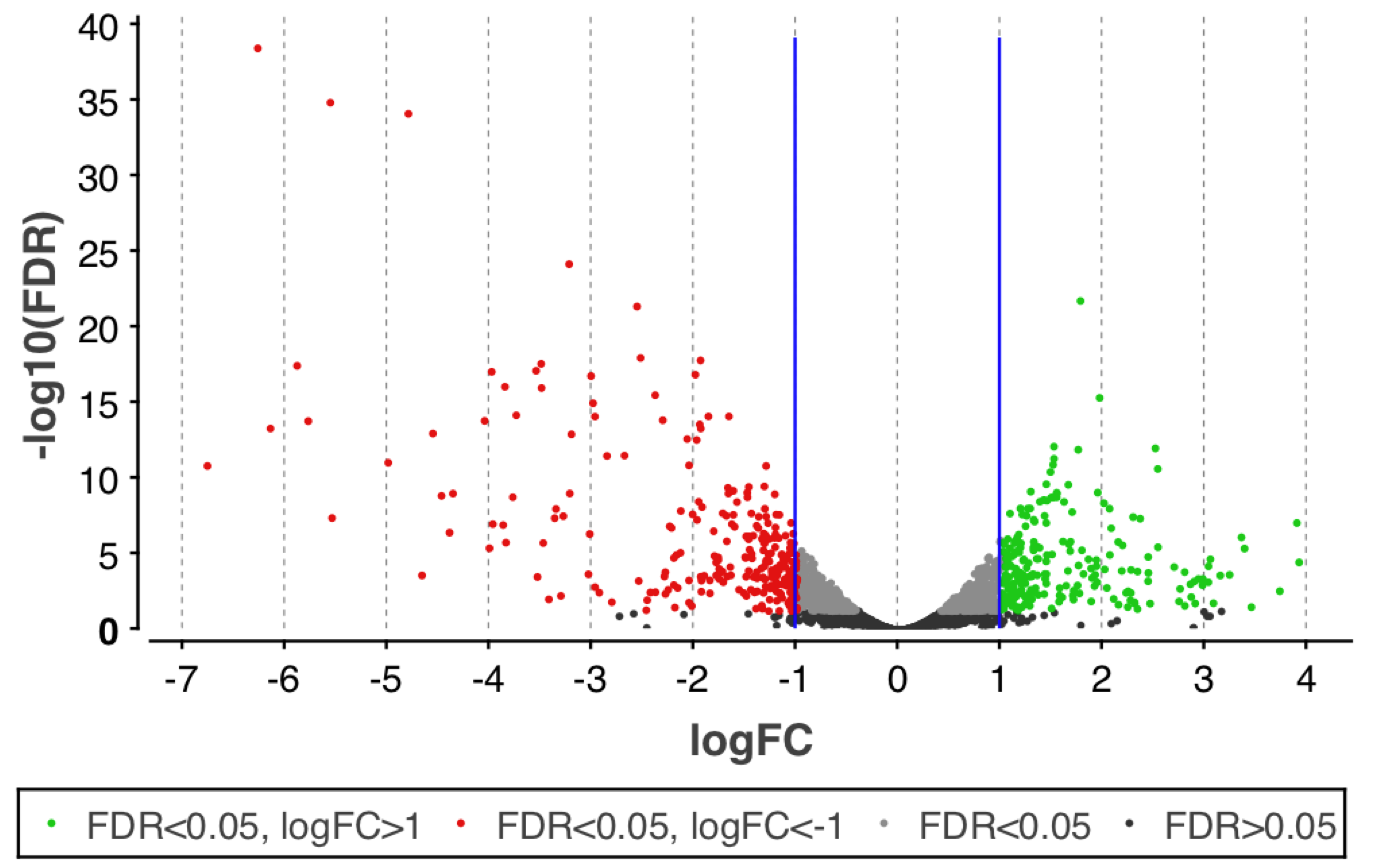

Supplement: S3 Fig — Volcano plot showing the overall differential gene transcription in bacteria cultured in SB vs. SB+M. Blast2GO was used to generate the volcano plot based on EdgeR values. (TIF) [file pone.0190599.s003.tif]

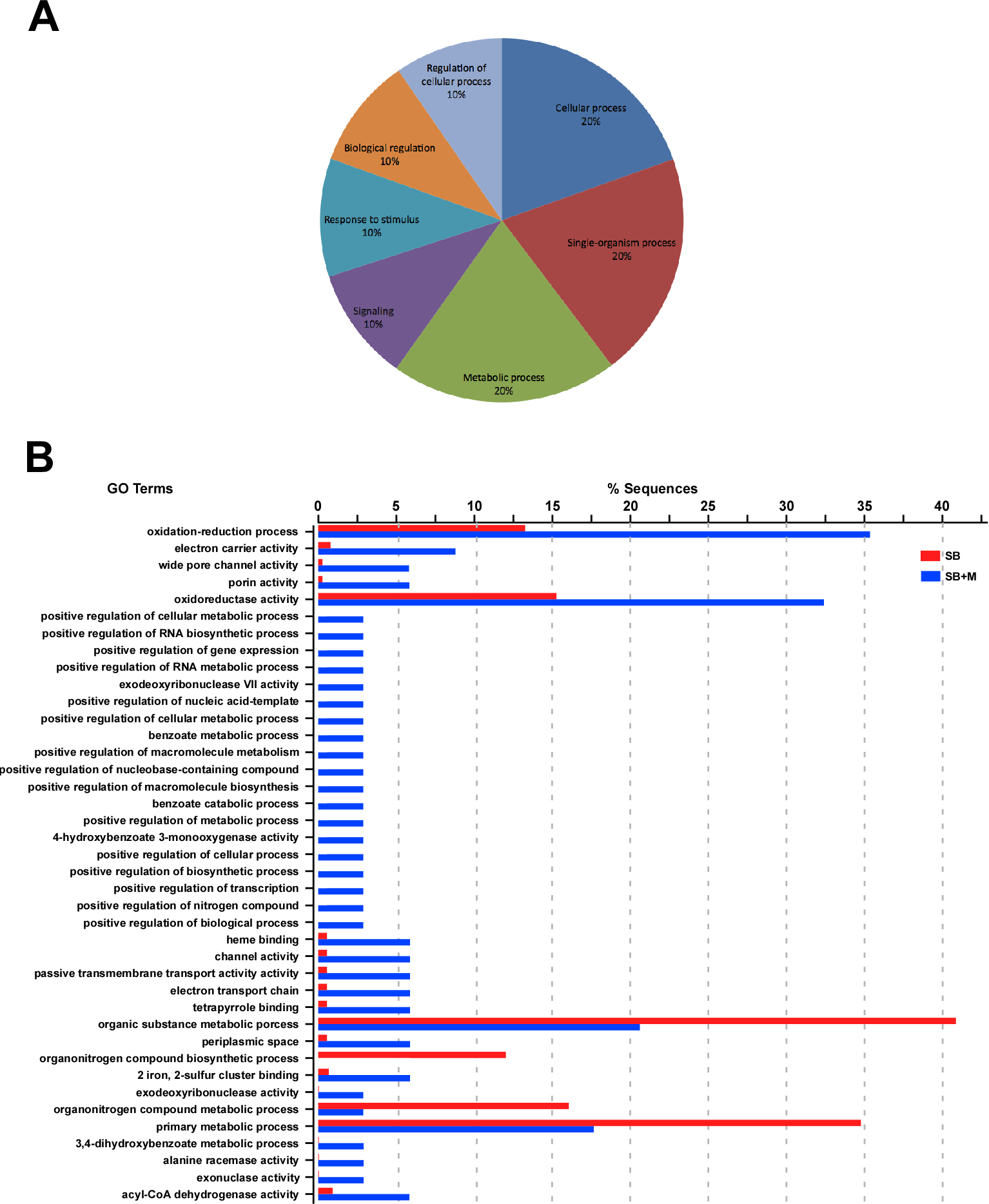

Supplement: S4 Fig — (A) Functional distribution of the 427 predicted protein-coding genes differentially transcribed in cells cultured in SB and SB+M. (B) Gene ontology (GO) analysis of the mucin-regulated genes using Blast2GO. (TIF) [file pone.0190599.s004.tif]
