# Supplementary material for: Mucin acts as a nutrient source and a signal for the differential expression of genes coding for cellular processes and virulence factors in Acinetobacter baumannii
Source: PLoS One. 2018 Jan 8;13(1):e0190599. doi: 10.1371/journal.pone.0190599 (PMC5757984; doi:10.1371/journal.pone.0190599)
Supplement: S1 Table — (DOCX) [file pone.0190599.s005.docx]

**S1 Table.**  **Primers used in this work.**

| Primer number | Gene target | Nucleotide sequence |
| --- | --- | --- |
| 3968 | *recA* | 5’-GCCCAGAAACTACCACTCG-3’ |
| 3969 | *recA* | 5’-GCTTCTTTAAACGGAGGAGCC-3’ |
| 3204 | *csuAB* | 5’-CAGGCTGTACTGTAGGTG-3’ |
| 3205 | *csuAB* | 5’-CAGGATCTGTTCCGTCAC-3’ |
| 3970 | *bauA* | 5’-GGAATGTATCGAGATGGAGATGC-3’ |
| 3971 | *bauA* | 5’-GTTAACACCACGTGTCACGC-3’ |
| 4402 | *benP* | 5’-CGGCGTAAACTGATAGCGCAGGT-3’ |
| 4403 | *benP* | 5’-CGGCTGTTGATGCTAGCCGCCAG-3’ |
| 4404 | *tssH* | 5’-GCCCCAGCTTGCAAAAGCCCCAT-3’ |
| 4405 | *tssH* | 5’-GCGCAGACGTCAGAACAACCCAA-3’ |
| 4406 | *paaH* | 5’-CCCGGAGTCGACTTGGTGAGTAC-3’ |
| 4407 | *paaH* | 5’-CGGCGGGCATTGCTCACCCT-3’ |
