# Supplementary material for: Mucin acts as a nutrient source and a signal for the differential expression of genes coding for cellular processes and virulence factors in Acinetobacter baumannii
Source: PLoS One. 2018 Jan 8;13(1):e0190599. doi: 10.1371/journal.pone.0190599 (PMC5757984; doi:10.1371/journal.pone.0190599)
Supplement: S2 Table — (DOCX) [file pone.0190599.s006.docx]

**S2 Table. Quality data collected from sequencing cDNA libraries constructed using RNA isolated from ATCC 19606^T^ cells cultured in SB or SB+M.**

| SB Library Sequencing Data | | | | | | | |
| --- | --- | --- | --- | --- | --- | --- | --- |
|  | Total count | Average count | Average % of reads | Average length | Total number of bases | Average number of bases | Average % of bases |
| Mapped reads | 43,409,618 | 14,469,873 | 97.27% | 74.01 | 3,212,537,602 | 1,070,845,867 | 97.28% |
| Not mapped reads | 1,223,718 | 407,906 | 2.73% | 73.76 | 90,223,877 | 30,074,626 | 2.72% |
| Reads in pairs | 39,391,740 | 13,130,580 | 88.35% | 124.9 | 2,913,223,008 | 971,074,336 | 88.30% |
| Broken paired reads | 4,017,878 | 1,339,293 | 8.92% | 74.49 | 299,314,593 | 99,771,531 | 8.98% |
| Total reads | 44,633,336 | 14,877,779 | 100.00% | 74 | 3,302,761,479 | 1,100,920,493 | 100.00% |
|  |  |  |  |  |  |  |  |
| SB+M Library Sequencing Data | | | | | | | |
|  | Total Count | Average count | Average % of reads | Average length | Total number of bases | Average number of bases | Average % of bases |
| Mapped reads | 47,757,110 | 15,919,037 | 98.83% | 74.34 | 3,550,367,191 | 1,183,455,730 | 98.84% |
| Not mapped reads | 563,520 | 187,840 | 1.17% | 73.78 | 41,580,144 | 13,860,084 | 1.16% |
| Reads in pairs | 43,156,168 | 14,385,389 | 89.24% | 132.56 | 3,207,737,013 | 1,069,245,671 | 89.23% |
| Broken paired reads | 4,600,942 | 1,533,647 | 9.59% | 74.46 | 342,630,178 | 114,210,059 | 9.61% |
| Total reads | 48,320,630 | 16,106,877 | 100.00% | 74.34 | 3,591,947,335 | 1,197,315,778 | 100.00% |
|  |  |  |  |  |  |  |  |
| Overall Library Sequencing Data | | | | | |  |  |
|  | Total count | Average % of reads | Average length | Total number of bases | Average % of bases |  |  |
| Mapped reads | 91,166,728 | 98.05% | 74.18 | 6,762,904,793 | 98.06% |  |  |
| Not mapped reads | 1,787,238 | 1.95% | 73.77 | 131,804,021 | 1.94% |  |  |
| Reads in pairs | 82,547,908 | 88.80% | 128.73 | 6,120,960,021 | 88.77% |  |  |
| Broken paired reads | 8,618,820 | 9.26% | 74.48 | 641,944,771 | 9.30% |  |  |
| Total reads | 92,953,966 | 100.00% | 74.17 | 6,894,708,814 | 100.00% |  |  |

All data were collected with Passing Filter with 98.3% >Q30
