# Supplementary material for: Mucin acts as a nutrient source and a signal for the differential expression of genes coding for cellular processes and virulence factors in Acinetobacter baumannii
Source: PLoS One. 2018 Jan 8;13(1):e0190599. doi: 10.1371/journal.pone.0190599 (PMC5757984; doi:10.1371/journal.pone.0190599)
Supplement: S3 Table — (DOCX) [file pone.0190599.s007.docx]

**S3 Table. *A. baumannii* ATCC 19606^T^ gene up-regulated by the presence of 0.5% mucin in swimming broth.**

| **Gene identifier** | **Fold change** | ***P* value** | **Predicted function** |
| --- | --- | --- | --- |
| A1S_0005 | 4.876856033 | 5.79461E-07 | Cytochrome b precursor |
| A1S_0009 | 4.535463545 | 1.22927E-05 | RND type efflux pump |
| prpB (A1S_0073)^§^ | 2.256823408 | 0.001232441 | 2-methylisocitrate lyase |
| A1S_0074 | 2.436213557 | 0.001448092 | 2-methylcitrate synthase/citrate synthase II family protein, pseudogene |
| A1S_0076 | 2.058803438 | 0.009488764 | Aconitate hydratase |
| A1S_0101 | 2.417121401 | 0.010172319 | Methylmalonate-semialdehyde dehydrogenase, pseudogene |
| A1S_0103 | 2.291988117 | 0.016345817 | 3-hydroxyisobutyrate dehydrogenase |
| A1S_0104 | 2.214320009 | 0.031303506 | Acetyl-CoA synthetase/AMP-(fatty) acid ligase |
| A1S_0105 | 2.217156233 | 0.01599743 | Acyl-CoA dehydrogenase |
| A1S_0106 | 2.557525827 | 0.00316474 | Enoyl-CoA hydratase/isomerase |
| A1S_0107 | 2.960278778 | 0.001415848 | Enoyl-CoA hydratase |
| A1S_0108 | 3.385075781 | 0.000404392 | Major facilitator superfamily metabolite/H(+) symporter |
| A1S_0109 | 2.005578327 | 5.83485E-05 | Homoserine lactone synthase |
| A1S_0177 | 2.268383958 | 8.24056E-07 | Cysteine synthase A/O-acetylserine sulfhydrolase A subunit PLP-dependent enzyme |
| A1S_0257 | 2.06453203 | 0.000165672 | Fe^2+^-trafficking protein |
| A1S_0258 | 2.533949935 | 2.43003E-05 | Argininosuccinate lyase |
| A1S_0279 | 2.000897325 | 0.021529666 | Elongation factor Tu |
| fadA (A1S_0305) | 2.153099142 | 0.006087876 | 3-ketoacyl-CoA thiolase, FadA |
| A1S_0449 | 3.995308158 | 1.37876E-08 | Coniferyl aldehyde dehydrogenase (CALDH) |
| clpX (A1S_0477) | 2.049805338 | 0.00307486 | ATPase and specificity subunit of ClpX-ClpP ATP-dependent serine protease |
| A1S_0486 | 2.297663715 | 2.61999E-06 | Gluconate kinase |
| A1S_0487 | 2.483135742 | 1.00234E-07 | Aldehyde dehydrogenase, pseudogene |
| A1S_0545 | 2.53432894 | 0.000147413 | Cetohydroxy acid isomeroreductase |
| A1S_0591 | 2.126336588 | 0.000478223 | Long-chain-fatty-acid-CoA ligase |
| A1S_0683 | 5.117058812 | 1.41461E-06 | Sigma(54) modulation protein RpoX, stress-response protein |
| A1S_0690 | 2.684637704 | 1.05547E-05 | Pilus subunit protein FilA |
| A1S_0754 | 2.187429899 | 0.003373818 | NADH:ubiquinone oxidoreductase subunit C/D |
| A1S_0755 | 2.307700992 | 0.001117444 | NADH dehydrogenase I E subunit E |
| A1S_0756 | 2.469437059 | 0.000653644 | NADH dehydrogenase I subunit F |
| A1S_0757 | 2.366086417 | 0.002066849 | NADH dehydrogenase subunit G |
| A1S_0758 | 2.287136558 | 0.003517374 | NADH dehydrogenase subunit H |
| A1S_0759 | 2.275271124 | 0.003174267 | NADH dehydrogenase subunit I |
| A1S_0761 | 2.403445757 | 0.001435073 | NADH dehydrogenase I subunit K |
| A1S_0774 | 2.423251713 | 7.24835E-09 | RND efflux transporter |
| A1S_0788 | 2.147185216 | 0.004609118 | Heme utilization protein |
| A1S_0820 | 3.153800409 | 8.08944E-05 | Peptidoglycan-binding LysM |
| A1S_0854 | 2.182759457 | 9.24588E-06 | NAD-dependent succinate aldehyde dehydrogenases |
| A1S_0855 | 2.096435486 | 4.06701E-05 | Dioxygenase subunit beta |
| A1S_0893 | 2.493875578 | 5.71885E-07 | RnfH protein family, member of the ubiquitin superfamily |
| A1S_0910 | 2.221622218 | 1.96052E-06 | Gamma-glutamyltranspeptidase |
| A1S_0952 | 2.211569592 | 0.014881584 | Major facilitator superfamily permease |
| A1S_0953 | 4.155106416 | 2.08256E-05 | Cupin domain protein, mannose-6-phosphate isomerase |
| A1S_0954 | 3.675535189 | 5.93865E-05 | Alpha/beta hydrolase family protein, CatD |
| A1S_0956 | 2.177325999 | 0.014618736 | L-aspartate dehydrogenase |
| A1S_0957 | 2.43728155 | 0.007415014 | Betaine-aldehyde dehydrogenase |
| A1S_0958 | 2.423328781 | 0.004294133 | Acetolactate synthase large subunit |
| A1S_1004 | 2.811858288 | 5.06913E-05 | Citrate transporter |
| A1S_1080 | 2.275389806 | 2.55235E-06 | NLPA lipoprotein |
| A1S_1086 | 2.049175749 | 6.2011E-05 | Calcium channels and CHEmotaxis (CACHE) receptor protein/Histidie kinase |
| A1S_1158 | 3.088908525 | 6.42461E-05 | Uncharacterized protein |
| A1S_1159 | 2.230561804 | 0.000683283 | Uncharacterized protein |
| A1S_1193 | 2.480689253 | 0.000532076 | OmpA/MotB protein |
| A1S_1205 | 2.164208074 | 0.008525197 | Alkyl hydroperoxide reductase C22 subunit, AhpC |
| A1S_1209 | 5.473142215 | 0.002552661 | Benzoate transport porin, BenP |
| A1S_1210 | 5.025999281 | 0.006720615 | Major facilitator superfamily transporter, BenK |
| A1S_1211 | 4.50743886 | 8.89275E-06 | Benzoate transporter, BenE |
| benD (A1S_1212) | 7.230360928 | 0.000872548 | 1,6-dihydroxycyclohexa-2,4-diene-1-carboxylate dehydrogenase, BenD |
| A1S_1213 | 6.923948731 | 0.003951293 | Benzoate 1,2-dioxygenase electron transfer component, BenC |
| A1S_1214 | 8.411393456 | 0.002683066 | Benzoate 1,2-dioxygenase subunit beta, BenB |
| A1S_1215 | 7.426423484 | 0.00201372 | Benzoate 1,2 dioxygenase subunit alpha, BenA |
| A1S_1261 | 2.350407725 | 1.15161E-08 | 3-hydroxyacyl-CoA dehydrogenase |
| A1S_1288 | 2.081708589 | 1.3823E-05 | Type VI secretion system protein, VGR-like protein, TssI |
| A1S_1289 | 2.020380724 | 4.1958E-05 | Type VI secretion system protein, VGR-like protein, TssI |
| A1S_1292 | 2.014662816 | 0.003396271 | Uncharacterized protein |
| A1S_1293 | 2.587613744 | 0.000203864 | Type VI secretion protein, TssB |
| A1S_1294 | 3.409115093 | 3.63899E-06 | Type VI secretion protein, TssB |
| A1S_1295 | 4.153898235 | 1.80402E-07 | Type VI secretion protein, TssC |
| A1S_1296 | 5.765082934 | 1.10125E-08 | Type VI secretion system effector protein Hcp1, TssD |
| A1S_1297 | 2.844802691 | 1.09366E-11 | Type VI secretion system lysozyme-like protein, TssE |
| A1S_1298 | 2.106834338 | 0.000245984 | Type VI secretion system protein ImpG, TssF |
| A1S_1299 | 3.805552399 | 1.07029E-10 | Type VI secretion system protein ImpG, TssF |
| A1S_1301 | 2.843789765 | 7.87924E-09 | Uncharacterized membrane protein |
| A1S_1302 | 2.434126602 | 7.89556E-08 | Type VI secretion protein, TssM |
| A1S_1303 | 2.246292519 | 2.32718E-05 | Type VI secretion protein, TssM |
| A1S_1304 | 2.055714347 | 0.000201054 | Type VI secretion protein, TagF |
| A1S_1305 | 2.178282322 | 8.78075E-06 | Type VI secretion protein, TagN |
| A1S_1306 | 2.483203275 | 4.37454E-05 | PAAR domain containing protein |
| A1S_1307 | 2.14514296 | 1.82021E-06 | Type VI secretion ATPase, TssH |
| A1S_1309 | 2.249010853 | 3.32742E-07 | Type VI secretion protein, TssK |
| A1S_1341 | 7.870503414 | 0.042039166 | Enoyl-CoA hydratase, PaaF |
| A1S_1342 | 8.861252528 | 0.039866679 | Epoxyphenylacetyl-CoA isomerase, PaaG |
| A1S_1343 | 10.85004468 | 0.023505204 | 3-hydroxyacyl-CoA dehydrogenase, PaaH |
| A1S_1344 | 13.15884402 | 0.005538184 | Beta-ketoadipyl CoA thiolase, PaaJ |
| A1S_1345 | 15.00769651 | 0.001207025 | Phenylacetate-CoA ligase, PaaK |
| A1S_1346 | 14.78059953 | 0.00014185 | Phenylacetate-CoA ligase, PaaK |
| A1S_1347 | 8.214779513 | 0.00066628 | Phenylacetic acid degradation operon repressor protein, PaaX |
| A1S_1348 | 10.13187857 | 3.69166E-05 | Phenylacetic acid degradation protein, PaaY |
| A1S_1349 | 5.64077843 | 5.33322E-05 | Thioesterase domain-containing protein, PaaI |
| A1S_1372 | 3.802051601 | 0.000345683 | Hydroxymethylglutaryl-CoA lyase, MvaB |
| A1S_1373 | 3.25732149 | 0.003228408 | 3-methylcrotonyl-CoA carboxylase subunit alpha |
| A1S_1374 | 3.345393954 | 0.002883219 | 3-methylglutaconyl-CoA hydratase |
| A1S_1375 | 3.012793835 | 0.01043401 | 3-methylcrotonyl-CoA carboxylase subunit beta |
| A1S_1376 | 3.238990899 | 0.004131094 | Isovaleryl-CoA dehydrogenase |
| A1S_1378 | 5.408398444 | 0.000219374 | Long chain fatty-acid CoA ligase |
| A1S_1379 | 3.767670502 | 0.000390434 | SAM-dependent methyltransferase |
| A1S_1380 | 5.770084898 | 1.78561E-06 | DcaP-like porin protein |
| A1S_1381 | 2.711658853 | 2.33232E-07 | GDSL-like Lipase |
| A1S_1443 | 2.130728923 | 0.007915063 | Taurine ATP-binding transport system component, TauB |
| A1S_1467 | 2.003213585 | 0.023546957 | Glutamate symport transmembrane protein, GltT/GltP |
| A1S_1490 | 2.962050012 | 0.001116133 | Glutamate/aspartate periplasmic-binding protein, GltI |
| A1S_1491 | 3.600540965 | 5.80563E-05 | Glutamate/aspartate transport permease protein, GltJ |
| A1S_1492 | 3.366102462 | 0.000230756 | Glutamate/aspartate transport permease protein, GltK |
| A1S_1493 | 3.441857707 | 9.51136E-05 | Glutamate/aspartate transport ATP-binding protein, GltL |
| A1S_1579 | 2.074811921 | 2.11371E-05 | Cell division protein (ZapE) |
| A1S_1610 | 2.081951258 | 2.45929E-05 | Zn-dependent metalloendopeptidase |
| A1S_1618 | 2.035758437 | 1.62449E-05 | Nucleoside-diphosphate sugar epimerase |
| A1S_1638 | 2.113682485 | 5.53444E-07 | Peptidylprolyl isomerase (PPIase) |
| A1S_1639 | 2.382712005 | 4.34602E-07 | Peptidylprolyl isomerase (PPIase) |
| A1S_1724 | 2.500841365 | 5.86511E-09 | Major facilitator superfamily, alpha-ketoglutarate permease |
| aspA (A1S_1726) | 3.880974387 | 5.38945E-14 | Aspartate ammonia-lyase, catabolic 3-dehydroquinate dehydratase |
| A1S_1738 | 2.492569024 | 0.00117945 | Transcriptional regulator |
| A1S_1814 | 2.401041687 | 9.93427E-06 | Bile acid:sodium symporter/arsenical resistance protein |
| A1S_1815 | 3.032423133 | 1.96739E-07 | Long-chain fatty acid-CoA ligase |
| A1S_1816 | 2.092615938 | 0.00042146 | Long-chain fatty acid transport protein |
| A1S_1817 | 3.361943473 | 5.24771E-12 | Acyl-CoA dehydrogenase |
| A1S_1818 | 3.216329497 | 6.00059E-09 | MaoC-like dehydratase |
| A1S_1819 | 2.906607377 | 4.13969E-10 | 3-hydroxyacyl-CoA dehydrogenase |
| A1S_1821 | 2.804324541 | 5.17674E-09 | Short chain dehydrogenase |
| A1S_1843 | 2.800652852 | 0.024512529 | Muconate cycloisomerase I, benzoate degradation, CatB |
| A1S_1844 | 4.792432953 | 0.001106926 | Muconolactone Delta-isomerase, CatC |
| A1S_1845 | 4.711557575 | 0.001143271 | Catechol 1,2-dioxygenase, CatA |
| A1S_1846 | 2.700134212 | 0.000836053 | 3-oxoadipate CoA-transferase subunit A, CatI/PacI |
| A1S_1847 | 5.388171769 | 0.000601207 | 3-oxoadipate CoA-transferase subunit B, CatJ/PcaJ |
| A1S_1849 | 4.014897458 | 0.000112128 | Beta-ketoadipyl CoA thiolase, CatF/PcaF |
| A1S_1850 | 3.251441888 | 0.005491257 | 3-oxoadipate enol-lactonase 2, CatD |
| A1S_1851 | 4.262244677 | 0.005406904 | Penicillin G amidase |
| A1S_1852 | 4.637517532 | 0.001480446 | Phenylacetaldehyde dehydrogenase, FeaB |
| A1S_1853 | 4.401870192 | 0.007821506 | Putative tynE |
| tynA (A1S_1854) | 4.69193766 | 0.009162041 | Tyramine oxidase, copper-requiring |
| A1S_1855 | 4.798216032 | 0.00017088 | Transcriptional regulator |
| A1S_1856 | 9.373208823 | 0.000696445 | *p*-hydroxyphenylacetate hydroxylase C1:reductase component |
| A1S_1857 | 6.695360404 | 0.006865368 | Vanillate O-demethylase oxidoreductase |
| A1S_1858 | 7.366319481 | 0.004179325 | Short-chain dehydrogenase |
| A1S_1859 | 7.579664989 | 0.003910183 | Aromatic-ring-hydroxylating dioxygenase small subunit |
| A1S_1860* | 8.113167493 | 0.004013873 | Aromatic-ring-hydroxylating dioxygenase large subunit, Rieske (2Fe-2S) protein |
| A1S_1861* | 7.792995922 | 0.006111932 | Aromatic-ring-hydroxylating dioxygenase large subunit, Rieske (2Fe-2S) protein |
| A1S_1862 | 8.794613566 | 0.004149482 | Polyketide cyclase |
| A1S_1863 | 7.81283848 | 0.010369188 | Catabolism of indolacetic acid, IacB |
| A1S_1864 | 7.182753897 | 0.011618012 | Acyl-CoA dehydrogenase-like protein, IacB-like protein |
| A1S_1865 | 6.909011585 | 0.049036233 | Glu-tRNA amidotransferase |
| A1S_1867 | 3.666570958 | 0.025285809 | Major facilitator superfamily transporter |
| A1S_1868* | 4.925893295 | 0.002549497 | Aromatic compound porin protein |
| A1S_1869* | 4.785364141 | 0.009283729 | Aromatic compound porin protein |
| A1S_1870 | 2.657842784 | 1.16991E-09 | Short-chain dehydrogenase |
| A1S_1871 | 2.174533709 | 3.93614E-06 | Phosphoglycerate mutase related protein |
| A1S_1872 | 2.090682651 | 2.80648E-06 | Phosphotransferase |
| A1S_1873 | 2.26895644 | 1.2012E-05 | Acyl-CoA dehydrogenase |
| A1S_1880 | 2.131415963 | 1.08363E-05 | Pyrroloquinoline-quinone QuiA |
| A1S_1882 | 2.305421626 | 1.20139E-05 | 3-dehydroshikimate dehydratase |
| aroD (A1S_1883) | 2.537667143 | 7.03524E-06 | 3-dehydroquinate dehydratase |
| A1S_1884 | 2.672152015 | 1.04452E-05 | Protocatechuate 3,4-dioxygenase subunit alpha, PcaG |
| A1S_1885 | 2.234148816 | 2.88521E-05 | Protocatechuate 3,4-dioxygenase subunit beta, PcaH |
| A1S_1886 | 2.556497961 | 2.75334E-07 | Gamma-carboxymuconolactone decarboxylase, PcaC |
| A1S_1887* | 2.275183296 | 8.0351E-06 | 4-hydroxybenzoate transporter, PcaK |
| A1S_1888* | 2.577267732 | 6.89506E-07 | 4-hydroxybenzoate transporter, PcaK |
| A1S_1891 | 3.816740734 | 0.000529673 | Beta-ketoadipyl CoA thiolase, PcaF |
| A1S_1892 | 5.012024813 | 0.000100525 | Beta-ketoadipyl CoA thiolase, PacF |
| A1S_1894 | 2.946424012 | 0.008872698 | 3-oxoadipate CoA-transferase subunit A, PcaI |
| A1S_1924 | 6.429886857 | 1.88258E-06 | Cytochrome d terminal oxidase polypeptide subunit I, CydA |
| A1S_1925 | 8.114881419 | 2.60992E-06 | Cytochrome d terminal oxidase polypeptide subunit II, CydB |
| A1S_1926 | 10.35799469 | 5.0383E-07 | Cyd operon YbgE family protein |
| A1S_1956 | 3.473051794 | 0.000481621 | Amino acid permease, AnsP |
| A1S_1957 | 3.740625425 | 0.006047654 | L-kynurenine hydrolase |
| A1S_2021 | 3.774833076 | 6.47997E-07 | Uncharacterized protein |
| A1S_2022 | 2.05653182 | 0.000308927 | Phage tail fiber protein |
| A1S_2044 | 2.055212821 | 6.48876E-05 | Ferredoxin-dependent glutamate synthase |
| A1S_2072 | 2.345968329 | 0.003610297 | Universal stress family protein |
| A1S_2111 | 2.113793669 | 0.000110462 | Dihydropteridine reductase |
| A1S_2130 | 2.048958463 | 1.21356E-06 | Translocation and assembly module TamB protein |
| A1S_2136 | 2.666483157 | 0.023871309 | Glutamine synthetase |
| A1S_2137 | 2.165033342 | 0.015439359 | Response regulator |
| A1S_2166 | 2.026413775 | 0.018869709 | Cytochrome bo(3) ubiquinol oxidase, CyoA |
| A1S_2196 | 2.150925848 | 0.000695212 | Membrane-associated dicarboxylate transport protein |
| A1S_2202 | 2.362610922 | 3.66085E-07 | Aspartate racemase |
| A1S_2213 | 2.715635166 | 1.95526E-10 | Pili protein, CsuE |
| A1S_2214 | 3.138205041 | 1.23081E-14 | Pili usher protein, CsuD |
| A1S_2215 | 3.047452851 | 1.09467E-08 | Pili assembly chaperone protein, CsuC |
| A1S_2218 | 2.152070566 | 0.012039198 | Pili protein, CsuA/B |
| A1S_2232 | 2.778516617 | 5.45342E-10 | Methylmalonate-semialdehyde dehydrogenase, MmsA |
| A1S_2248 | 2.476314797 | 6.57746E-08 | 2-keto-D-gluconate reductase |
| A1S_2296 | 3.178133365 | 6.37313E-08 | Collagenase-like protease |
| A1S_2308 | 2.183985812 | 3.11462E-06 | Triacylglycerol lipase |
| A1S_2338 | 2.321699725 | 0.000396794 | Malate dehydrogenase, MaeB |
| A1S_2348 | 3.192409387 | 2.16553E-10 | Triglyceride lipase |
| A1S_2416 | 2.004972195 | 0.023842263 | Uncharacterized protein |
| A1S_2449 | 5.39149804 | 0.003395911 | Aromatic amino acid APC transporter, AroP |
| A1S_2450 | 4.909586525 | 0.043281158 | Pyruvate decarboxylase |
| A1S_2452 | 3.094142173 | 0.00193887 | Betaine-aldehyde dehydrogenase |
| A1S_2475 | 2.285469911 | 0.002414222 | Isocitrate dehydrogenase, Icd |
| A1S_2532 | 2.842882752 | 0.004772248 | Sulfate transport protein |
| A1S_2538 | 2.091909892 | 0.011128303 | Outer membrane protein CarO precursor |
| A1S_2589 | 4.081294884 | 4.20755E-06 | Aminoacylase-2/carboxypeptidase-Z family hydrolase, AbgB |
| A1S_2595 | 2.322001399 | 0.00099752 | Peptidoglycan-associated lipoprotein, outer membrane protein P6 OmpA/MotB |
| A1S_2601 | 2.435095049 | 2.38221E-05 | CSLREA domain outer membrane protein, putative outer membrane protein A |
| A1S_2619 | 2.07721379 | 0.000207433 | RND efflux transporter |
| A1S_2620 | 2.674043431 | 1.09622E-08 | RND efflux transporter, HlyD secretion family protein |
| A1S_2633 | 2.703468173 | 0.000182712 | D-alanine/D-serine/glycine transport protein |
| groEL (A1S_2664) | 2.004304238 | 0.007905263 | 60-kDa chaperonin, GroEL |
| A1S_2672 | 2.705395329 | 5.51688E-05 | Uncharacterized protein |
| A1S_2696 | 2.411595888 | 0.00240277 | Type I secretion protein |
| A1S_2702 | 2.830055349 | 3.73942E-08 | 1,3-propanediol dehydrogenase |
| sucA (A1S_2715) | 2.048840719 | 0.007128676 | 2-oxoglutarate dehydrogenase |
| A1S_2716 | 2.19742636 | 0.003297483 | 2-oxoglutarate dehydrogenase, SucB |
| A1S_2717 | 2.23412058 | 0.002492584 | Dihydrolipoamide dehydrogenase |
| A1S_2718 | 2.207779293 | 0.002958372 | Succinyl-CoA synthetase subunit beta, SucC |
| A1S_2719 | 2.16669819 | 0.003352092 | Succinyl-CoA synthetase subunit alpha, SucD |
| A1S_2753 | 3.668933839 | 4.00203E-05 | DcaP-like porin protein |
| A1S_2758 | 2.900317398 | 3.05491E-08 | Membrane protease subunit stomatin/prohibitin-like protein |
| A1S_2763 | 2.206786121 | 0.000130274 | Aromatic amino acid APC transporter |
| A1S_2773 | 2.336819778 | 0.004592853 | Long-chain fatty acid transport protein |
| A1S_2809 | 2.099989642 | 0.015747164 | Bacteriolytic lipoprotein entericidin B |
| A1S_2820 | 2.164270029 | 0.007378672 | Uncharacterized protein |
| A1S_2860 | 2.704676827 | 1.55308E-08 | MFS transporter, metabolite:H+ symporter (MHS) family protein |
| A1S_2880 | 2.217008163 | 4.01132E-07 | META domain uncharacterized protein |
| A1S_3001 | 2.09340248 | 0.010479864 | 30S ribosomal protein S9 |
| A1S_3049 | 2.512531821 | 0.001475156 | TRAP transporter, DctM-like membrane protein |
| A1S_3050 | 2.36586316 | 0.002020538 | Rhomboid family peptidase |
| rpmJ (A1S_3060) | 2.704393986 | 0.000271796 | 50S ribosomal protein L36 |
| rplB (A1S_3077) | 2.200490858 | 0.011652471 | 50S ribosomal protein L2 |
| rplW (A1S_3078) | 2.335538398 | 0.006746206 | 50S ribosomal subunit protein L23 |
| rplD (A1S_3079) | 2.077175016 | 0.02674767 | 50S ribosomal subunit protein L4 |
| rplC (A1S_3080) | 2.228349746 | 0.014304529 | 50S ribosomal protein L3 |
| A1S_3099 | 2.198292751 | 0.001249414 | toluene-tolerance protein, Ttg2E; sulphate transporter and anti-sigma factor antagonist |
| A1S_3108 | 4.197963643 | 3.75571E-07 | Oxygen-dependent coproporphyrinogen-III oxidase (HemF) |
| A1S_3113 | 2.670054933 | 0.006787703 | Aminotransferase, class III domain protein |
| A1S_3160 | 4.406299142 | 2.84633E-06 | Lipase |
| A1S_3178 | 2.902145915 | 2.08018E-09 | Uncharacterized protein |
| A1S_3179 | 2.830602312 | 3.88568E-08 | Uncharacterized protein |
| A1S_3303 | 2.043993391 | 0.002154566 | Uncharacterized protein |
| A1S_3350 | 2.037048239 | 0.025343682 | Sigma 54 modulation protein/S30EA ribosomal protein |
| A1S_3431 | 2.794875576 | 0.000116299 | Histidine triad (HIT) family protein, hydrolase |
| A1S_3458 | 2.069772433 | 0.00761301 | Membrane transporter protein |

^§^A1S_numbers represent cognate gene identifiers if these were annotated as the rest of the predicted genomic coding regions.
