# Supplementary material for: Mucin acts as a nutrient source and a signal for the differential expression of genes coding for cellular processes and virulence factors in Acinetobacter baumannii
Source: PLoS One. 2018 Jan 8;13(1):e0190599. doi: 10.1371/journal.pone.0190599 (PMC5757984; doi:10.1371/journal.pone.0190599)
Supplement: S4 Table — (DOCX) [file pone.0190599.s008.docx]

**S4 Table. *A. baumannii* ATCC 19606^T^ gene down-regulated by the presence of 0.5% mucin in swimming broth.**

| **Gene identifier** | **Fold change** | ***P* value** | **Predicted function** |
| --- | --- | --- | --- |
| A1S_0012 | -2.707755881 | 4.59648E-05 | Epoxide hydrolase-like protein |
| A1S_0023 | -2.043365539 | 0.017535276 | C4-dicarboxylate transporter, malic acid transport protein, tellurite resistance protein |
| A1S_0030 | -2.263065333 | 0.021904089 | ABC transported, alkanesulfonate transport protein |
| A1S_0078 | -2.07089818 | 0.004083241 | Uncharacterized protein |
| A1S_0089 | -2.221574238 | 0.004916182 | RNA pseudouridine synthase |
| A1S_0095 | -2.626624867 | 0.000419808 | D-amino acid dehydrogenase |
| A1S_0096 | -2.10271674 | 0.000236099 | Alanine racemase |
| A1S_0114 | -2.676653406 | 0.008223817 | Phosphopantetheine attachment domain protein, acyl carrier protein |
| A1S_0126 | -2.163069963 | 6.99418E-05 | LysE-type translocator family protein |
| A1S_0166 | -2.458738083 | 0.006737144 | DNA protecting protein, DprA |
| A1S_0180 | -2.070208144 | 0.006737093 | Uncharacterized membrane protein |
| A1S_0204 | -2.214803937 | 0.042518777 | Class II aldolase/adducin domain-containing protein |
| A1S_0224 | -3.976612495 | 5.13441E-16 | Uncharacterized protein |
| A1S_0234 | -2.176247998 | 0.000189765 | Type IV fimbriae expression response regulatory protein, PilR |
| A1S_0256 | -4.692975883 | 7.76338E-12 | High affinity phosphate uptake transcriptional repressor, PhoU |
| A1S_0296 | -2.249057005 | 0.028142223 | Selenocysteine synthase |
| A1S_0300 | -2.109322246 | 0.000114458 | Transcriptional regulator, MerR family |
| A1S_0311 | -2.38644371 | 0.000758037 | Acyl-CoA thioesterase |
| A1S_0437 | -2.318253611 | 7.47132E-07 | MarR family transcriptional regulator |
| A1S_0462 | -4.856772439 | 4.37285E-05 | Phosphatase |
| A1S_0463 | -21.07140251 | 3.86471E-14 | Alkaline phosphatase |
| A1S_0464 | -22.0927977 | 9.75421E-17 | Twin arginine-targeting protein translocase, TatC |
| A1S_0465 | -58.68193174 | 1.34097E-27 | Twin arginine-targeting protein translocase, TatB |
| A1S_0466 | -108.5300229 | 2.06179E-21 | Twin arginine-targeting protein translocase, TatA |
| A1S_0494 | -15.90672696 | 7.54937E-11 | Glycosyl transferase |
| A1S_0495 | -2.425927645 | 0.002326186 | Glycosyltransferase, group 1 family protein |
| A1S_0619 | -2.502004375 | 1.91858E-08 | Carbon-nitrogen hydrolase |
| A1S_0663 | -3.846591185 | 8.85469E-08 | Replication protein RepA |
| A1S_0664 | -4.961431988 | 4.34688E-07 | Replication protein RepC |
| A1S_0665 | -3.855474361 | 2.36077E-15 | Conjugal transfer protein TrbJ |
| A1S_0666 | -2.756510942 | 0.000115864 | Plasmid conjugal transfer protein TrbL/VirB6 |
| A1S_0673 | -2.108788061 | 0.0003709 | Transposase |
| A1S_0691 | -2.608847369 | 5.51035E-05 | Peptidase C39 family protein |
| A1S_0724 | -2.427584478 | 0.001644274 | Citrate utilization protein B |
| A1S_0736 | -3.198291907 | 3.96832E-06 | Uncharacterized protein |
| A1S_0739 | -4.127982593 | 0.049406884 | TetR family transcriptional regulator |
| A1S_0741 | -4.588082085 | 3.43241E-06 | Unknown function Darcynin protein |
| A1S_0743 | -2.65376667 | 0.043784136 | Unknown function Darcynin protein |
| A1S_0770 | -3.179855404 | 1.71026E-13 | Uncharacterized protein |
| A1S_0773 | -2.481925325 | 0.002333575 | D-cysteine desulfhydrase, DcyD |
| A1S_0789 | -2.005957363 | 0.005475624 | tRNA-dihydrouridine synthase |
| A1S_0796 | -2.124297382 | 0.000109761 | GntR family transcriptional regulator |
| A1S_0804 | -2.257043565 | 0.032172165 | Trehalose 6-phosphate phosphatase |
| A1S_0826 | -2.38591258 | 9.61671E-07 | Peptidyl-tRNA hydrolase |
| ipk (A1S_0833)^§^ | -3.08834413 | 0.000360485 | 4-diphosphocytidyl-2C-methyl-D-erythritol kinase |
| A1S_0889 | -15.64026093 | 1.54134E-14 | GNAT family acetyltransferase |
| A1S_0976 | -2.578479897 | 3.58159E-05 | Formylglycine-generating sulfatase enzyme family protein |
| A1S_0980 | -2.113680918 | 5.00265E-05 | Ferric enterobactin outer membrane receptor protein FepA |
| A1S_0981 | -3.385434836 | 5.21485E-06 | Ferric enterobactin outer membrane receptor protein FepA |
| A1S_1029 | -2.628301414 | 3.36013E-05 | Ribosomal RNA large subunit methyltransferase H |
| A1S_1044 | -14.41769701 | 7.52715E-17 | Co/Zn/Cd efflux system |
| A1S_1045 | -6.442313497 | 2.08596E-12 | Co/Zn/Cd efflux system |
| A1S_1046 | -4.072974219 | 1.07545E-09 | LysE-type translocator family protein |
| A1S_1063 | -2.016852776 | 0.002267793 | TonB-dependent siderophore receptor protein |
| A1S_1081 | -2.32719023 | 0.034197452 | TetR family transcriptional regulator |
| A1S_1116 | -2.41605734 | 4.64102E-05 | Vanillate O-demethylase oxygenase subunit protein Van A |
| A1S_1123 | -2.235978562 | 0.000609163 | 4-hydroxyacetophenone monooxygenase |
| A1S_1131 | -3.20332817 | 0.00012645 | Metal-dependent hydrolase |
| A1S_1132 | -2.471933569 | 0.00092634 | Oxidoreductase short chain dehydrogenase/reductase family protein |
| A1S_1197 | -7.96283415 | 2.32299E-07 | Extracellular nuclease |
| A1S_1198 | -10.9053525 | 3.66214E-09 | Extracellular nuclease |
| A1S_1220 | -2.435880934 | 8.06518E-08 | LysE-type translocator family protein |
| A1S_1229 | -2.016340261 | 0.000184616 | Pyrroline-5-carboxylate reductase |
| A1S_1253 | -2.152306283 | 0.004096671 | EamA-like transporter family protein |
| A1S_1319 | -3.503176932 | 7.03994E-07 | Uncharacterized protein |
| A1S_1350 | -2.781814283 | 0.00230405 | TetR family transcriptional regulator |
| A1S_1353 | -2.075814602 | 0.027788227 | LysR family transcriptional regulator |
| A1S_1359 | -23.11843364 | 3.44387E-19 | ABC-type Fe^3+^ transport system, periplasmic component |
| A1S_1360 | -16.17581269 | 2.57193E-16 | ABC-type Fe^3+^ transport system, periplasmic component |
| A1S_1361 | -3.784028714 | 0.000130653 | ABC transporter, ATP-binding protein |
| A1S_1362 | -4.83973109 | 0.000161892 | ABC transporter permease, 2-aminoethylphosphonate transport, Fe^3+^ transport |
| A1S_1364 | -3.001619643 | 2.38345E-08 | 2-aminoethylphosphonate-pyruvate transaminase |
| A1S_1384 | -3.764800371 | 1.60469E-06 | Competence-induced protein, CinA |
| A1S_1390 | -4.220986768 | 9.91882E-08 | Amino acid efflux transmembrane protein |
| A1S_1396 | -2.799442102 | 3.65477E-05 | Amino acid ABC transporter |
| A1S_1407 | -2.090124428 | 0.036495615 | Serine acetyltransferase |
| A1S_1408 | -3.231272061 | 2.36551E-06 | Rhodanese-related sulfurtransferase |
| A1S_1447 | -2.146226422 | 0.039221337 | Permease for cytosine/purine, uracil, thiamine, allantoin family protein |
| A1S_1453 | -3.946626277 | 1.5421E-10 | ArsR family transcriptional regulator |
| A1S_1463 | -2.05144196 | 0.000100131 | Serine O-acetyltransferase |
| A1S_1472 | -3.368268645 | 0.003652007 | Amino acid efflux protein, putative threonine efflux protein |
| A1S_1477 | -2.040161315 | 0.000322498 | Cytosine/purine, uracil, thiamine, allantoin permease family protein |
| A1S_1497 | -2.279706908 | 0.004319339 | Putative acyltransferase |
| A1S_1507 | -3.172539576 | 2.79166E-07 | Pili protein |
| A1S_1508 | -2.087214148 | 0.002362008 | Pili biogenesis outer membrane usher protein |
| A1S_1509 | -2.504789041 | 6.55813E-05 | Pili biogenesis outer membrane chaperone protein |
| A1S_1510 | -2.217489548 | 8.57204E-05 | Pili subunit protein |
| A1S_1515 | -2.256180623 | 0.001199901 | Transcriptional regulator |
| A1S_1553 | -2.317106774 | 8.65984E-07 | MotA/TolQ/ExbB proton channel protein, ExbB |
| A1S_1647 | -2.724272435 | 0.000509833 | Baumannoferrin biosynthesis protein, BfnA |
| A1S_1648 | -3.276727264 | 0.000359948 | Lysine/ornithine N-monooxygenase, baumannoferrin biosynthesis protein, BfnB |
| A1S_1649 | -2.798094895 | 0.002457074 | RND efflux transporter, baumannoferrin export protein, BfnC |
| A1S_1655 | -4.491350383 | 2.53649E-05 | Ferric siderophore receptor protein, baumannoferrin receptor protein, BfmH |
| A1S_1662 | -2.941837975 | 0.000264374 | Histidine phosphatase super family protein |
| A1S_1666 | -2.121147589 | 0.011632395 | Membrane peptidase |
| A1S_1667 | -2.101551062 | 6.59999E-05 | Ferric hydroxamate siderophore receptor protein, FhuE |
| A1S_1669 | -2.065280018 | 0.000539352 | TetR family transcriptional regulator |
| A1S_1677 | -54.81176443 | 7.14717E-21 | TetR family transcriptional regulator |
| A1S_1766 | -4.781993245 | 4.07828E-06 | Uncharacterized protein |
| A1S_1767 | -14.40445994 | 8.01189E-11 | Survival protein SurE-like phosphatase |
| A1S_1768 | -2.562212424 | 0.009733046 | Uncharacterized protein |
| A1S_1769 | -2.361020813 | 0.034197452 | RND efflux membrane protein |
| A1S_1773 | -3.035528323 | 0.000144243 | RND efflux membrane protein |
| A1S_1774 | -2.194362066 | 0.036495615 | Mandelamide hydrolase |
| A1S_1785 | -2.036033809 | 0.000581524 | Iron ABC transport protein |
| A1S_1803 | -2.704955526 | 0.020513199 | Uncharacterized membrane protein |
| A1S_1808 | -2.147094448 | 0.013055011 | Dicarboxylate carrier protein |
| A1S_1823 | -2.012885674 | 3.90815E-05 | TetR family transcriptional regulator |
| A1S_1825 | -2.267514431 | 1.17678E-05 | Rrf2 family transcriptional regulator |
| A1S_1928 | -11.75425098 | 8.20176E-30 | Uncharacterized protein |
| A1S_1933 | -3.37355768 | 0.000119921 | Uncharacterized protein |
| A1S_1934 | -2.259820166 | 0.000289139 | Uncharacterized protein |
| A1S_1944 | -2.221167162 | 9.40378E-06 | Alpha/beta hydrolase |
| A1S_1988 | -2.390679702 | 0.002502816 | Sulfur relay protein TusD/DsrE |
| A1S_1989 | -3.464122505 | 8.32576E-08 | Uncharacterized protein |
| A1S_2006 | -2.514879896 | 0.029367388 | ANTAR-domain response regulator |
| A1S_2055 | -2.046325513 | 0.001057985 | Phospholipase C |
| A1S_2067 | -2.789374401 | 5.26849E-08 | AsnC family transcriptional regulator |
| A1S_2069 | -7.689129605 | 1.35076E-05 | MgtC Mg^2+^ family transporter |
| A1S_2070 | -10.84105677 | 2.27148E-05 | MgtA Mg^2+^ ABC transporter ATPase |
| A1S_2075 | -2.139600202 | 0.001501313 | OmpW family protein |
| A1S_2076 | -2.290492952 | 0.000388929 | TonB-dependent siderophore receptor protein, FhuE |
| A1S_2080 | -2.460606915 | 0.000179307 | TonB-dependent siderophore receptor, Cir family protein, CirA |
| A1S_2083 | -2.305753174 | 1.48437E-07 | AsnC family transcriptional regulator |
| A1S_2091 | -2.824975357 | 1.74537E-07 | Pili subunit protein |
| A1S_2103 | -2.442644272 | 0.006737144 | Ethanolamine transporter |
| A1S_2118 | -2.543160805 | 6.52603E-08 | Addiction module antitoxin, RelB/DinJ family protein |
| A1S_2123 | -2.005592811 | 0.016755214 | Uncharacterized protein |
| A1S_2151 | -2.041963971 | 0.002282639 | AraC family transcriptional regulator |
| A1S_2156 | -2.092941202 | 0.000775335 | LysR substrate-binding domain regulatory protein |
| A1S_2224 | -2.469850504 | 7.70616E-06 | LysE-type translocator family protein |
| A1S_2317 | -2.010688473 | 0.028057981 | Rare lipoprotein A |
| A1S_2376 | -3.48002397 | 0.000866488 | ABC transport protein, acinetobactin secretion protein, BarA |
| A1S_2378 | -2.056743703 | 0.000471467 | ABC transport protein, acinetobactin secretion protein, BarB |
| A1S_2379 | -2.471048652 | 0.014106234 | Acinetobactin biosynthesis protein, BasG |
| A1S_2380 | -3.043297793 | 0.029873076 | Acinetobactin biosynthesis protein, BasF |
| A1S_2386 | -2.638073309 | 0.000870159 | Ferric acinetobactin periplasmic binding protein, BauB |
| A1S_2387 | -4.349789327 | 0.002566617 | Ferric acinetobactin transport protein, BauE |
| A1S_2396 | -2.037228148 | 0.040884164 | TetR family transcriptional regulator |
| A1S_2420 | -2.432386068 | 1.9964E-07 | EF-P beta-lysylation protein EpmB |
| A1S_2445 | -3.416948938 | 8.53187E-07 | Phosphate import ATP-binding protein PstB |
| A1S_2446 | -13.40837622 | 8.60938E-21 | Phosphate transport system permease protein PstA |
| A1S_2447 | -15.87820244 | 9.27043E-19 | Phosphate transport system permease protein |
| A1S_2448 | -32.11558164 | 2.20625E-19 | Phosphate transport system substrate-binding protein |
| A1S_2473 | -2.771566398 | 1.24534E-05 | LysR family transcriptional regulator |
| A1S_2512 | -2.254387891 | 7.51618E-07 | Uncharacterized protein |
| A1S_2598 | -2.471744528 | 0.000675631 | RNA polymerase sigma-70 factor |
| A1S_2647 | -2.783447427 | 0.000665462 | TetR family transcriptional regulator |
| A1S_2651 | -2.008222614 | 0.014768451 | Non-ribosomal peptide synthetase module-containing protein |
| A1S_2653 | -2.495162803 | 6.77615E-08 | Transcription elongation factor GreB |
| A1S_2661 | -3.591995675 | 0.000398075 | Glycerophosphodiester phosphodiesterase |
| A1S_2677 | -3.809656159 | 0.000110621 | Alkaline phosphatase D family protein |
| A1S_2709 | -2.067176894 | 0.000540716 | Uncharacterized protein |
| A1S_2734 | -2.332109776 | 0.000346108 | Phosphatidylglycerophosphatase, PAP2 family protein |
| A1S_2744 | -4.231260886 | 0.000784821 | SAM-dependent methyltransferase |
| A1S_2749 | -3.021754039 | 0.002186855 | 2 phosphatidic acid phosphatase, PAP2 family protein |
| A1S_2807 | -2.087988534 | 2.04998E-05 | Uncharacterized inner membrane protein |
| A1S_2816 | -2.069603458 | 0.022961837 | Uncharacterized protein |
| A1S_2821 | -2.467338722 | 4.09571E-05 | Alkylphosphonate uptake protein PhnA |
| A1S_2822 | -2.155111828 | 0.000156883 | Uncharacterized protein |
| A1S_2830 | -2.056190769 | 3.32035E-06 | 3-methyladenine DNA glycosylase |
| A1S_2855 | -2.541816453 | 6.01264E-07 | Uncharacterized protein |
| A1S_2985 | -2.309884285 | 3.27261E-05 | Uncharacterized protein |
| A1S_3012 | -2.329453563 | 6.25278E-07 | Acyl-CoA dehydrogenase |
| A1S_3014 | -2.005800327 | 0.000117412 | S-(Hydroxymethyl)glutathione synthase |
| A1S_3044 | -3.366730139 | 0.000571031 | HAD phosphoserine phosphatase-like hydrolase, IB family protein |
| A1S_3048 | -3.092680681 | 1.64293E-05 | Uncharacterized small metal-binding protein |
| A1S_3053 | -2.363316602 | 0.000538256 | Acyl-CoA dehydrogenase |
| A1S_3105 | -2.068259954 | 0.000311094 | Inositol monophosphatase |
| A1S_3112 | -2.423537481 | 3.44469E-08 | Uncharacterized protein |
| A1S_3115 | -2.209705832 | 0.013113047 | DNA metabolism protein |
| A1S_3146 | -2.630531682 | 3.10962E-05 | MFS transporter, DHA1 family, multidrug/chloramphenicol efflux transporter |
| gmk (A1S_3170) | -2.020885179 | 4.47802E-06 | Guanylate kinase Gmk |
| A1S_3174 | -5.393271807 | 0.000347121 | Bacterioferritin-associated ferredoxin |
| A1S_3193 | -2.66908389 | 0.003965884 | Pilus assembly protein PilO |
| A1S_3207 | -2.109587828 | 2.6177E-05 | Sulfate ABC transporter, sulfate-binding protein |
| A1S_3224 | -3.873975766 | 4.14034E-12 | Fatty acyl-CoA reductase |
| A1S_3239 | -2.126402765 | 0.000162108 | Cro/Cl family transcriptional regulator |
| A1S_3264 | -2.568577037 | 0.004021269 | TetR family transcriptional regulator |
| A1S_3284 | -2.764160385 | 5.96644E-07 | DNA-binding helix-turn-helix protein |
| A1S_3285 | -5.860875223 | 5.24282E-13 | Branched-chain amino acid ABC transporter permease |
| A1S_3305 | -2.681233912 | 2.27727E-05 | FMN reductase |
| A1S_3323 | -2.097371544 | 0.000113864 | Metallo-beta-lactamase superfamily protein |
| A1S_3326 | -2.792622862 | 3.83934E-08 | Uncharacterized membrane protein |
| A1S_3339 | -2.168086854 | 0.036495615 | Ferrichrome-iron receptor |
| A1S_3341 | -3.906609557 | 7.77744E-12 | Exodeoxyribonuclease III |
| A1S_3374 | -3.202616004 | 5.02815E-06 | Phosphate regulon transcriptional regulatory protein PhoB |
| A1S_3375 | -5.249102289 | 9.56402E-11 | Phosphate regulon transcriptional regulatory protein PhoB |
| A1S_3376 | -2.212153424 | 1.97198E-06 | Phosphate regulon sensor kinase PhoR |
| A1S_3392 | -2.471553373 | 1.51708E-06 | Phosphatidylglycerophosphatase A |
| A1S_3410 | -2.007309253 | 0.004160703 | Acyltransferase |
| A1S_3444 | -2.036326135 | 8.88003E-06 | Uncharacterized membrane protein |
| A1S_3468 | -4.17174269 | 1.49912E-06 | pAB1 plasmid, Cro/Cl family transcriptional regulator |
| A1S_3469 | -7.895532961 | 1.20532E-09 | pAB1 plasmid, putative toxin-antitoxin system toxin component |
| A1S_3471 | -2.382917767 | 1.06925E-05 | pAB1 plasmid, initiator RepB protein |

^§^A1S_numbers represent cognate gene identifiers if these were annotated as the rest of the predicted genomic coding regions.
